# Atomically Dispersed Cobalt on Ionic Carbon Nitrides for Selective and Efficient Nitrate Electroreduction to Ammonia

**DOI:** 10.1002/anie.2543286

**Published:** 2026-03-09

**Authors:** Nana Gao, Minjuan Guo, Haijian Tong, Guoyu Hou, Jingwen Ba, Xiaoyu Zhang, Ruixin Zhang, Hui Zhang, Xianwei Fu, Leonardo Cancellara, Nadezda V. Tarakina, Yu Zhang, Tianxi Liu, Christian Mark Pelicano, Zhihong Tian

**Affiliations:** ^1^ Engineering Research Center For Nanomaterials Henan University Kaifeng P.R. China; ^2^ Department of Colloid Chemistry Max Planck Institute of Colloids and Interfaces Potsdam Germany; ^3^ School of Mechanical and Power Engineering East China University of Science and Technology Shanghai P.R. China; ^4^ Key Laboratory of Synthetic and Biological Colloids Ministry of Education School of Chemical and Material Engineering Jiangnan University Wuxi P.R. China

**Keywords:** electrocatalysis, NH_3_ synthesis, nitrate reduction, PHI, single‐atoms

## Abstract

Direct electrochemical conversion of nitrate to ammonia (NH_3_) represents a sustainable route for NH_3_ production while simultaneously mitigating nitrate pollution. Carbon nitrides (CNs) have emerged as promising supports for transition‐metal single‐atom catalysts due to their high nitrogen content and abundant coordination sites. However, conventional CNs generally suffer from poor electrical conductivity and difficulty in stabilizing high densities of atomically dispersed metal centers, which limits catalytic efficiency and selectivity in the nitrate reduction reaction. Herein, we overcome these limitations by constructing cobalt poly(heptazine imides) (*Co*PHI), an ionic carbon nitride in which Co^2+^ species are coordinated to negatively charged imide‐bridging nitrogen atoms. This coordination environment enables a high density of isolated Co active sites (1.092 wt.%) while enhancing charge transport through the PHI framework. As a result, *Co*PHI achieves a Faradaic efficiency of 93.5% and an NH_3_ yield rate of 46.1 mg·h^−1^·mg_cat._^−1^ at −0.8 V versus RHE, outperforming conventional Co─N─C and *Co*─C_3_N_4_ systems. Combined experimental and theoretical studies show that *Co*PHI promotes strong nitrate adsorption, facilitates water dissociation to supply protons, and stabilizes key intermediates, collectively enabling efficient and selective nitrate‐to‐ammonia conversion.

## Graphical abstract

By exploiting the structural benefits of PHI, we synthesized *Co*PHI with atomically dispersed cobalt sites at high density. This architecture enables efficient nitrate ion adsorption, promotes water dissociation, and enhances *H generation on the catalyst surface, thereby significantly improving electrochemical nitrate reduction performance.

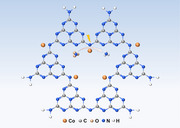

## 1 Introduction

Ammonia (NH_3_) is vital for fertilizer production and serves as a key intermediate in pharmaceuticals, refrigeration, and cleaning products [1, 2]. Industrial NH_3_ synthesis is dominated by Haber–Bosch process, which requires high temperatures (350–500°C) and pressures (150–350 bar). This energy‐intensive process accounts consumes 2% of global energy and contributes nearly 1% of global CO_2_ emissions [3, 4]. Electrochemical NH_3_ production from nitrate (NO_3_^−^) presents a promising alternative, combining safer conditions with the added benefit of reducing nitrate pollution from industrial and agricultural sources [5, 6, 7]. When powered by renewable electricity, the electrocatalytic NO_3_^−^ reduction reaction (NO_3_RR) can convert waste nitrogen into valuable NH_3_. However, the process involves complex proton‐coupled electron transfer steps, leading to limited efficiency and selectivity. Moreover, the competing H_2_ evolution reaction (HER) consumes electrons and suppresses NH_3_ formation [8, 9]. Hence, developing highly selective, cost‐effective and efficient electrocatalysts is necessary to advance this technology.

Transition metal single‐atom catalysts (TM SACs), anchored on N‐doped carbonaceous frameworks (TM–N_x_ *_‐_*), have emerged as efficient and cost‐effective catalysts for various electrocatalytic applications [10, 11, 12, 13, 14]. The N coordination sites modulate the local electronic environment of the metal centers, thereby enhancing the adsorption and activation of key reaction intermediates. However, in these metal–N_x_–C systems, the quantity, spatial distribution, and specific coordination environments of nitrogen dopants in the carbon matrix are often difficult to precisely control [15, 16]. To address these challenges, carbon nitrides (CNs) present a compelling alternative as support matrix for TM–N_x_ catalysts due to their high N content and structurally well‐defined coordination sites [17, 18], offering numerous anchoring points for metal atoms. However, early CNs have inherent poor conductivity and limited electrochemical stability which restrict their direct application in electrocatalysis. So far, only a few efforts have directly employed metal‐doped CNs in NO_3_RR, but these have generally yielded subpar performance compared to conventional systems [19, 20, 21].

In this regard, ionic CNs, such as metal poly(heptazine imides) (denoted as *M*PHI, with *M* being a metal cation), offer a particularly attractive platform. In these materials, metal cations are coordinated to negatively charged imide‐bridging N atoms, forming well‐defined metal‐N coordination environments within an ordered layered framework [22, 23]. This ionic character not only stabilizes isolated metal single atoms at high density, but also enhances electrical conductivity relative to conventional CNs, facilitating efficient charge transport during electrocatalysis. In addition, the PHI framework exhibits intrinsic ion‐exchange capability, allowing a variety of metal ions to be introduced either during synthesis or post‐synthetically [24, 25]. Although *M*PHIs possess favorable properties, their application support for NO_3_RR remains rare, presenting a promising yet underdeveloped research direction.

In this work, cobalt poly(heptazine imides) (*Co*PHI) were synthesized, with metal salt (CoCl_2_·6H_2_O) additionally introduced to the eutectic KCl–LiCl melt during PHI preparation. The negatively charged nitrogen atoms surrounding the imide bridges in the PHI layer facilitate the uniform incorporation of cobalt single atoms, enabling the successful synthesis of *Co*PHI with high‐density atomically dispersed Co active sites (1.092 wt.%). The *Co*PHI exhibits a high selectivity toward NH_3_, achieving a Faradaic efficiency (FE) of 93.5% and an NH_3_ yield rate of 46.1 mg·h^−1^·mg_cat._^−1^ at −0.8 V versus RHE. Both experimental and theoretical calculations confirm that the N‐coordination modulation strategy can effectively regulate the adsorption energy of NO_3_RR intermediates on Co metal centers, thereby accelerating the NO_3_RR reaction.

## 2 Results and Discussion

### 2.1 Catalyst Characterization

**FIGURE 1:**
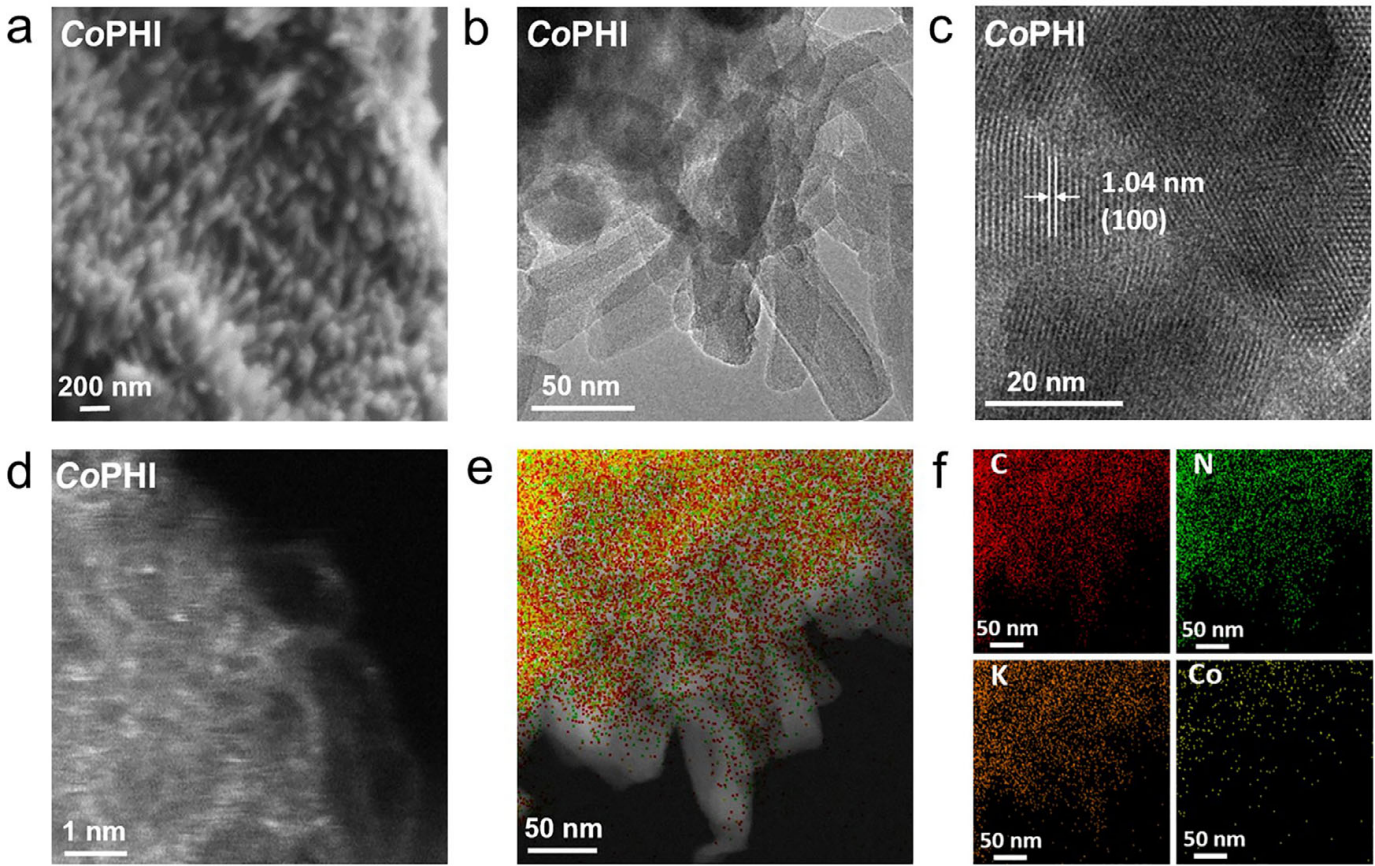
Characterizations of *Co*PHI catalysts. (a) SEM image, (b) TEM image, (c) HRTEM image, (d) AC HAADF‐STEM image, and (e) TEM image and (f) the corresponding elemental mapping images of C, N, K, Co in the *Co*PHI.

The synthetic route for preparing *Co*PHI is shown in Figure S1, where Co ions are incorporated into the PHI framework through a one‐step thermal polymerization carried out in KCl/LiCl eutectic salt melt, using CoCl_2_·6H_2_O (0.1 wt.%) as the Co precursor. As a control, pristine *K*PHI was synthesized following the same procedure, but without the inclusion of CoCl_2_·6H_2_O. The metal composition of the as‐prepared *Co*PHI and *K*PHI was analyzed by inductively coupled plasma optical emission spectrometry (ICP‐OES). *Co*PHI contained 1.092 wt.% cobalt, while its K content was lower than that of *K*PHI (Table S1), indicating that Co^2+^ partially substitutes K^+^ within the PHI framework. Unless otherwise stated, *Co*PHI hereafter refers to *Co*PHI‐0.1, which contains approximately 1.092 wt.% Co. The morphology of the synthesized catalysts was analyzed using scanning electron microscopy (SEM). Both *Co*PHI and *K*PHI displayed similar rod‐like structures (Figures 1a and S2). The transmission electron microscopy (TEM) image of the sample *Co*PHI is presented in Figure 1b, while high‐resolution TEM (HRTEM) images revealed clear lattice fringes with an interplanar spacing of 1.01 ± 0.05 and 0.31 ± 0.05 nm, which correspond to the (100) and (002) planes of the PHI, respectively (Figures 1c and S3a). Furthermore, aberration‐corrected high‐angle annular dark‐field scanning transmission electron microscopy (AC HAADF‐STEM) was employed to probe the dispersion of Co. As shown in Figure 1d, the presence of abundant bright spots corresponding to individual Co atoms provided direct evidence of their atomic dispersion within the *Co*PHI matrix. Further confirmation that the bright spots originate from Co single atoms rather than K species is provided by quantitative HAADF‐STEM intensity profiling and electron‐beam stability analysis (Figure S3b–d). Elemental mapping via TEM imaging further confirmed the uniform distribution of C, N, K, and Co throughout the catalyst (Figure 1e–f).

**FIGURE 2:**
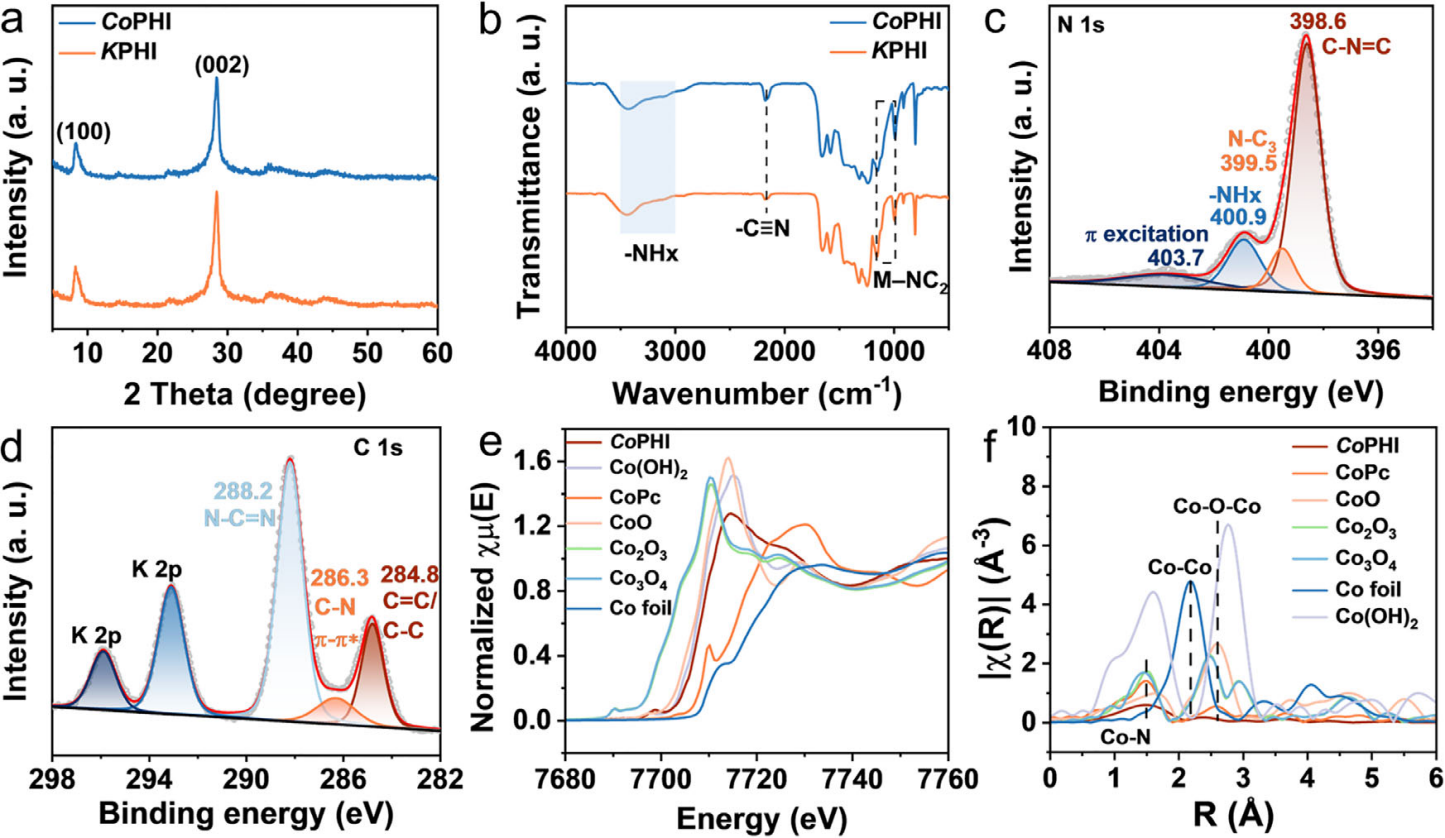
Structure of the catalysts. (a) XRD patterns and (b) FT‐IR spectra of samples. (c) N 1s and (d) C 1s XPS spectrum of *Co*PHI. (e) Co K‐edge XANES spectra, (f) the corresponding EXAFS spectra at k3‐weighted R‐space of *Co*PHI and reference samples (Co foil, CoPc, CoO, Co_2_O_3_, Co_3_O_4_ and Co(OH)_2_).

In addition, by maintaining all other synthesis conditions unchanged, we introduced 0.05 and 0.2 wt.% of CoCl_2_·6H_2_O to the KCl–LiCl salt template to prepare *Co*PHI with different cobalt contents. The resulting *Co*PHI‐0.05 and *Co*PHI‐0.2 samples contain 0.483 and 1.842 wt.%, respectively (Figure S4). As shown in Figures 2a and S5, the overall structural integrity remains largely preserved following the addition of CoCl_2_·6H_2_O during PHI synthesis. The sharp and intense diffraction peak at 8° in the XRD pattern of *Co*PHI corresponds to the (100) plane, indicative of the ordered repetition of heptazine units within the framework. Meanwhile, the peak at 28° is assigned to the (002) crystal plane, which reflects the interlayer stacking of the heptazine‐based layers [26, 27]. Complementary structural insights are provided by the Fourier‐transform infrared (FT‐IR) spectra (Figures 2b and S6), which show similar bonding environment across the samples. A broad absorption band between 3000 and 3500 cm^−1^ is observed, corresponding to terminal amino groups [28, 29]. Additionally, distinct peaks near 2180 cm^−1^ are attributed to the stretching modes of cyano groups (─C≡N) [30]. The absorption bands at 1640 and 1570 cm^−1^ are assigned to the vibrations of protonated primary amine (H_2_NC) and secondary amine (HNC_2_) groups, respectively, signifying the presence of both terminal and bridging N functionalities in the structure. Additional vibrational features within 1450–1254 cm^−1^ range correspond to typical modes of heteroaromatic systems, particularly the stretching and bending vibrations of C─N and C═N bonds, characteristic of heptazine framework [31, 32]. Notably, two distinct signals at 1150 and 997 cm^−1^ appear in all samples, which are attributed to metal coordination with N‐rich bridging sites (M─NC_2_; where M denotes a metal ion), suggesting incorporation of metal species into the PHI structure. The peak at 807 cm^−1^ is related to the out‐of‐plane breathing mode of heptazine rings [33].

XPS measurements were conducted to compare the elemental composition and electronic structure of *Co*PHI and *K*PHI. The absence of Li signals in the survey spectra of both materials (Figures S7a and S8a) confirms that Li was not incorporated into the PHI framework. As shown in Figure S7b, the XPS Co 2p spectrum of *Co*PHI exhibits a main peak centered at 780.7 eV along with an intense satellite feature at 787.0 eV, indicating that cobalt is in a divalent oxidation state in the material [34]. The high‐resolution N 1s spectrum displays four resolved components: a peak at 398.6 eV corresponds to sp^2^‐hybridized N atoms within the heptazine ring (C─N═C); a secondary peak at 399.5 eV is linked to tertiary N species (N─C_3_); the signal at 400.9 eV is indicative of amino‐type N (─NH_x_); and a high‐binding energy peak at 403.7 eV is related to surface charging effects (Figure 2c) [29, 33, 35]. Furthermore, the high‐resolution C 1s spectrum displays three distinct peaks (Figure 2d). The signal at 288.2 eV originates from sp^2^‐hybridized carbon in the N─C═N linkages of the aromatic heptazine core. A signal at 286.3 eV is assigned to C─NH_x_ groups. A secondary peak at 284.8 eV is commonly attributed to adventitious carbonaceous contamination, likely introduced during sample handling or from the ambient environment [36, 37].

To elucidate the electronic configuration and local coordination of Co species in *Co*PHI, x‐ray absorption spectroscopy was carried out. Comparative spectra were acquired from standard references such as metallic Co foil, cobalt oxides (CoO, Co_2_O_3_, and Co_3_O_4_), Co(OH)_2_ and cobalt phthalocyanine (CoPc, Figure S9). In the Co K‐edge x‐ray absorption near‐edge structure (XANES) spectrum, the absorption edge of *Co*PHI is found to fall between those of Co foil and Co(OH)_2_, indicating that the oxidation state of Co lies between 0 and +2 (Figures 2e,S10). The corresponding Fourier‐transformed extended x‐ray absorption fine structure (FT‐EXAFS) spectrum for *Co*PHI revealed a single pronounced peak at ∼1.5 Å, attributed to Co–N coordination, with no detectable Co–Co scattering at 2.18 Å or Co–O–Co contributions around 2.6 Å. This absence of higher‐order coordination features confirms the isolated, atomically dispersed nature of Co centers within the PHI matrix (Figure 2f). Importantly, the Co–N peak intensity in *Co*PHI was lower than that observed for CoPc, which is known to feature a CoN_4_ coordination motif. This suggests that Co atoms in *Co*PHI adopt a lower coordination environment than the square‐planar configuration in CoPc (Table S2) [38, 39].

### 2.2 Electrochemical NO_3_RR Performance

**FIGURE 3:**
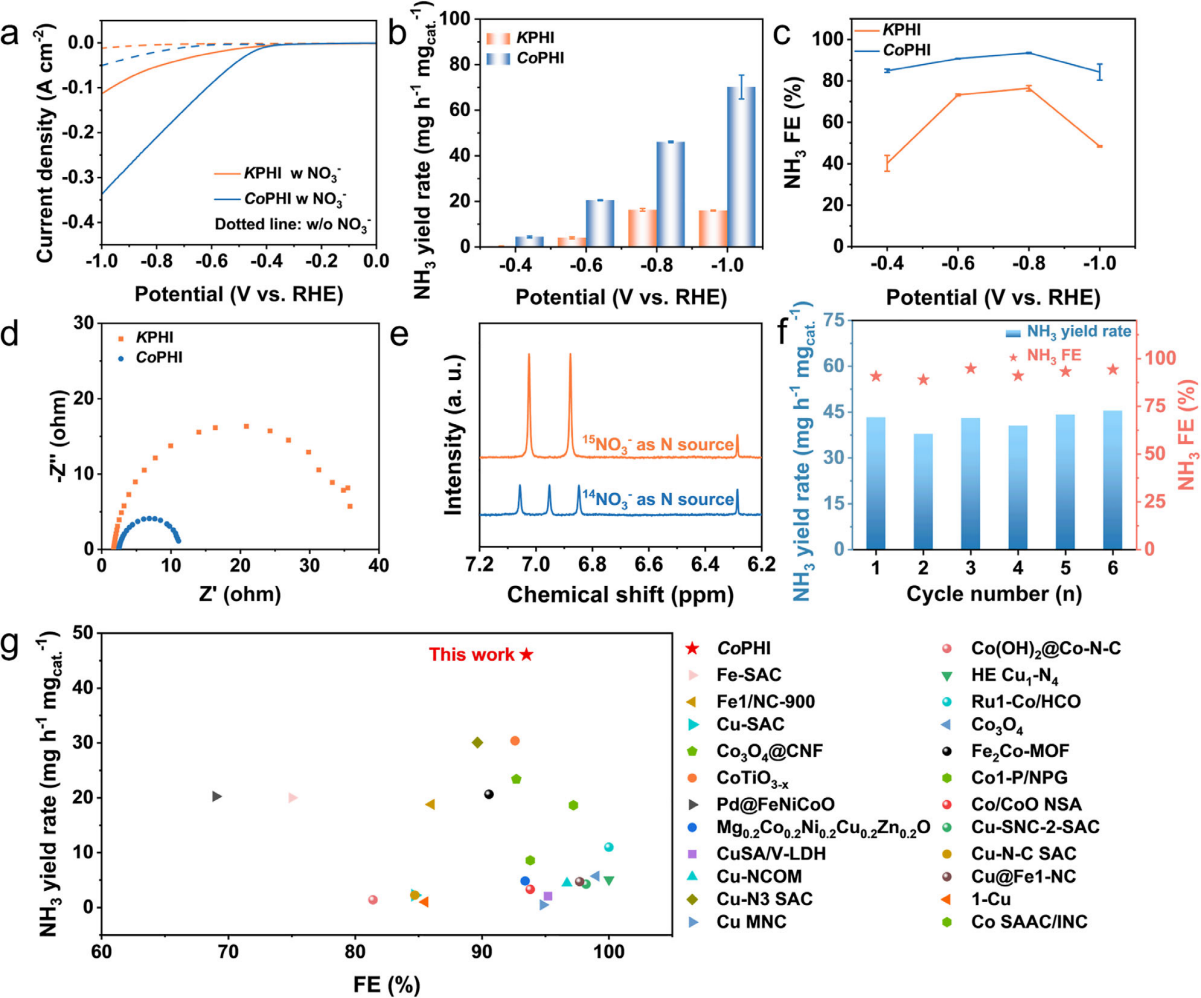
NO_3_RR performance. (a) LSV curves of *Co*PHI and *K*PHI in 0.1 M KOH with (solid line) or without (dotted line) 1.0 M KNO_3_. (b) NH_3_ yield rate and (c) FE at different potentials. (d) EIS curves of *Co*PHI and *K*PHI at −0.4 V versus RHE. (e) ^1^H NMR spectra of the electrolytes produced from the NO_3_RR using ^15^NO_3_^−^ and ^14^NO_3_^−^ standard samples. (f) Stability of electrochemical NO_3_RR of *Co*PHI at −0.8 V versus RHE for six times. (g) Comparisons of *Co*PHI with the reported electrocatalysts in terms of NO_3_RR performance.

The NO_3_RR performance of *Co*PHI was evaluated using a standard three‐electrode set‐up in an H‐type cell, with *K*PHI serving as a reference. To assess the intrinsic catalytic activity, 1.0 M of KNO_3_ in 0.1 M KOH was employed as the electrolyte. Linear sweep voltammetry (LSV) was conducted to analyze catalytic behavior toward NO_3_RR, while UV–vis spectrophotometry was used to quantify the concentrations of NH_3_ and nitrite (NO_2_^−^) via a colorimetric assay (Figure S11) (please see Supporting Information for detailed experimental procedures). Initially, *Co*PHI was tested in 0.1 M KOH electrolyte both with and without nitrate. The presence of nitrate led to a substantial increase in current density at the same applied potential, as evidence by the LSV curves (Figure 3a). Specifically, at −1.0 V versus RHE, *Co*PHI achieved a current density of 337 mA cm^−2^ in nitrate‐containing electrolyte, compared to just 50 mA cm^−2^ in nitrate‐free KOH solution. The sharp contrast highlights that NO_3_RR is far more favorable than the competing HER on *Co*PHI. Overall, *Co*PHI demonstrated significantly enhanced response in KNO_3_/KOH relative to pure KOH solution, confirming that the majority of the observed current stems from nitrate reduction. Across the entire potential range tested, *Co*PHI outperformed *K*PHI, reaching a peak current density of 337 mA cm^−2^ at −1.0 V versus RHE.

NH_3_ yield is a key metric for evaluating the efficiency of NO_3_RR. Since the production rate is directly influenced by current density, NH_3_ quantification was performed under galvanostatic conditions (Figure S12). As shown in Figure 3b,c, *Co*PHI consistently delivers higher Faradaic efficiency (FE) for NH_3_ generation across all tested potentials compared to *K*PHI. Notably, at −0.8 V versus RHE, *Co*PHI achieves a peak NH_3_ FE of 93.5%, with a corresponding production rate of approximately 46.1 mg h^−1^ mg_cat._^−1^. These findings verify that *Co*PHI shows superior NO_3_RR activity relative to *K*PHI, evidenced by both increased NH_3_ yield rate and FE. *K*PHI likely possesses an insufficient number of active sites to further reduce the NO_2_^−^ intermediates, resulting in a significantly higher NO_2_^−^ yield rate and FE compared to *Co*PHI (Figure S13). The *Co*PHI catalysts with varying cobalt loadings were systematically evaluated for NO_3_RR, revealing that *Co*PHI‐0.1 delivers the optimal catalytic performance (Figure S14). The results indicate that appropriate Co loading is crucial for achieving maximal NO_3_RR activity. To further clarify the distinct role of Co sites in *Co*PHI, we conducted poisoning experiments by introducing thiocyanate ions (SCN^−^) into the electrolyte to selectively deactivate the Co sites. A pronounced decrease in current density, along with reduced NH_3_ yield rate and FE, was observed for *Co*PHI during NO_3_RR (Figures S15a and S16). As a comparison, *K*PHI showed negligible decay (Figure S15b). These results demonstrate the critical role of Co sites in the catalytic process. In addition, we further evaluated their nitrite reduction reaction (NO_2_RR) performance (Figure S17). Compared to *K*PHI, *Co*PHI shows a comparable NO_2_RR activity (Figure S18) but a significantly enhanced NO_3_RR performance, revealing that Co sites effectively promote the conversion of NO_3_^−^ to NO_2_^−^. Furthermore, NO_2_RR measurements were carried out on poisoned *K*PHI and *Co*PHI. A sharp decrease in the NO_2_RR current density was observed for *Co*PHI after poisoning, whereas *K*PHI exhibited negligible changes (Figure S19). These findings confirm that the enhanced activity primarily originates from the intrinsic Co active sites. To understand the origin of this enhanced activity, the electrochemical active surface area (ECSA) of each catalyst was evaluated via double‐layer capacitance (*C*_dl_) measurements [40], derived from cyclic voltammetry within the non‐Faradaic region (Figure S20a and b). As depicted in Figure S20c, the estimated *C*_dl_ of *Co*PHI is 296.85 µF cm^−2^, significantly surpassing that of *K*PHI (161.14 µF cm^−2^), indicating a larger ECSA and greater availability of active sites [41]. Furthermore, electrochemical impedance spectroscopy (EIS) was used to compare the charge transfer behavior of the catalysts (Figure 3d). The Nyquist plots reveal that *Co*PHI exhibits lower charge transfer resistance than *K*PHI, suggesting more efficient interfacial electron transport [42]. Under the optimal FE condition of *Co*PHI (−0.8 V versus RHE), the NH_3_ yield in the electrolyte was further quantified by ^1^H nuclear magnetic resonance (NMR) spectroscopy (Figure S21), revealing a NH_3_ yield rate of 46.1 mg h^−1^ mg_cat._^−1^ and an FE of 90.7%. These results are consistent with those obtained by UV–vis spectrophotometry (46.08 mg h^−1^ mg_cat._^−1^, 90.6%, Figure S22). To verify that NH_3_ is indeed produced from NO_3_RR, a ^1^H isotopic labeling experiment was carried out to confirm that NO_3_^−^ serves as the sole N source. When ^14^NO_3_^−^ was used, the resulting ^14^NH_4_^+^ exhibited a characteristic triplet signal in the ^1^H NMR spectrum at 6.84, 6.92, and 7.01 ppm. In contrast, employing ^15^NO_3_^−^ as the N source resulted in a distinct doublet for ^15^NH_4_^+^ at 6.86 and 6.98 ppm, thereby confirming nitrate origin of the NH_3_ (Figure 3e) [43]. Besides, blank experiments were conducted in the 0.1 M KOH solution without nitrate, and no NH_3_ was detected (Figure S23), thereby excluding nitrogen interference originating from the electrocatalyst itself or the external environment [44, 45]. Additionally, *Co*PHI maintains high NH_3_ yield rate and FE over six consecutive cycles, underscoring its excellent electrochemical stability and durability (Figure 3f). Compared to the control samples and recently reported electrocatalysts for NO_3_RR, *Co*PHI exhibits higher activity (Figure 3g and Table S3). Post‐reaction characterizations of *Co*PHI were carried out using XRD (Figure S24a), FT‐IR (Figure S24b), ICP (Table S1), and XPS (Figure S25). After electroreduction, the XRD peak intensity of *Co*PHI decreased but remained visible, indicating that the PHI framework was largely preserved. Consistently, no significant changes were observed in the N 1s, C 1s XPS spectra, or FTIR spectra, further indicating structural stability. Meanwhile, an increased intensity of the Co 2p signal, together with the unchanged oxidation state and nearly identical Co content (1.088 wt.%), suggests that the accumulation of Co^2^^+^ species at the catalyst surface played a role in facilitating the NO_3_RR [34]. Additionally, the concentration of Co ions leached into the electrolyte during NO_3_RR was quantified by inductively coupled plasma‐mass spectrometry (ICP‐MS). The detected Co concentration is 0.00442 µg mL^−1^, accounting for only 0.04 wt.% of the catalyst mass, which is negligible, thus confirming the excellent structural stability of *Co*PHI and excluding contributions from homogenous Co species.

To assess the inherent advantages of the PHI structure, we also synthesized bulk C_3_N_4_ for Co single‐atom immobilization as a reference. The XRD patterns of C_3_N_4_ and *Co–*C_3_N_4_ display two diffraction peaks at 13.2° and 27.4°, corresponding to the (100) and (002) planes of C_3_N_4_, which are attributed with repeated heptazine units and interlayer stacking of conjugated aromatic systems, respectively (Figure S26) [46, 47]. Moreover, no reflections attributable to metallic Co are detected in *Co–*C_3_N_4_ sample. FT‐IR spectra of both materials also exhibit nearly identical absorption features, including vibrations of ─OH/─NH_x_ groups, CN heterocycles, and out‐of‐plane bending modes of the heptazine ring (Figure S27) [32, 48, 49]. These results confirm that incorporation of Co single atoms does not alter the chemical framework of C_3_N_4_. ICP‐OES analysis further revealed a Co content of 0.94 wt.% in *Co–*C_3_N_4_ sample (Table S1), comparable to that of *Co*PHI. However, electrocatalytic testing shows that *Co–*C_3_N_4_ is markedly less active for NO_3_RR than *Co*PHI (Figures S28–S30), achieving only an NH_3_ yield rate of 967 mg h^−1^ mg_Co_^−1^ with a FE of 52.3% at −0.8 V versus RHE. EIS demonstrates that *Co*PHI exhibits a lower charge‐transfer resistance than *Co–*C_3_N_4_ (Figure S31), underscoring the superior conductivity of the PHI structure relative to conventional CNs.

### 2.3 Mechanism Investigation

**FIGURE 4:**
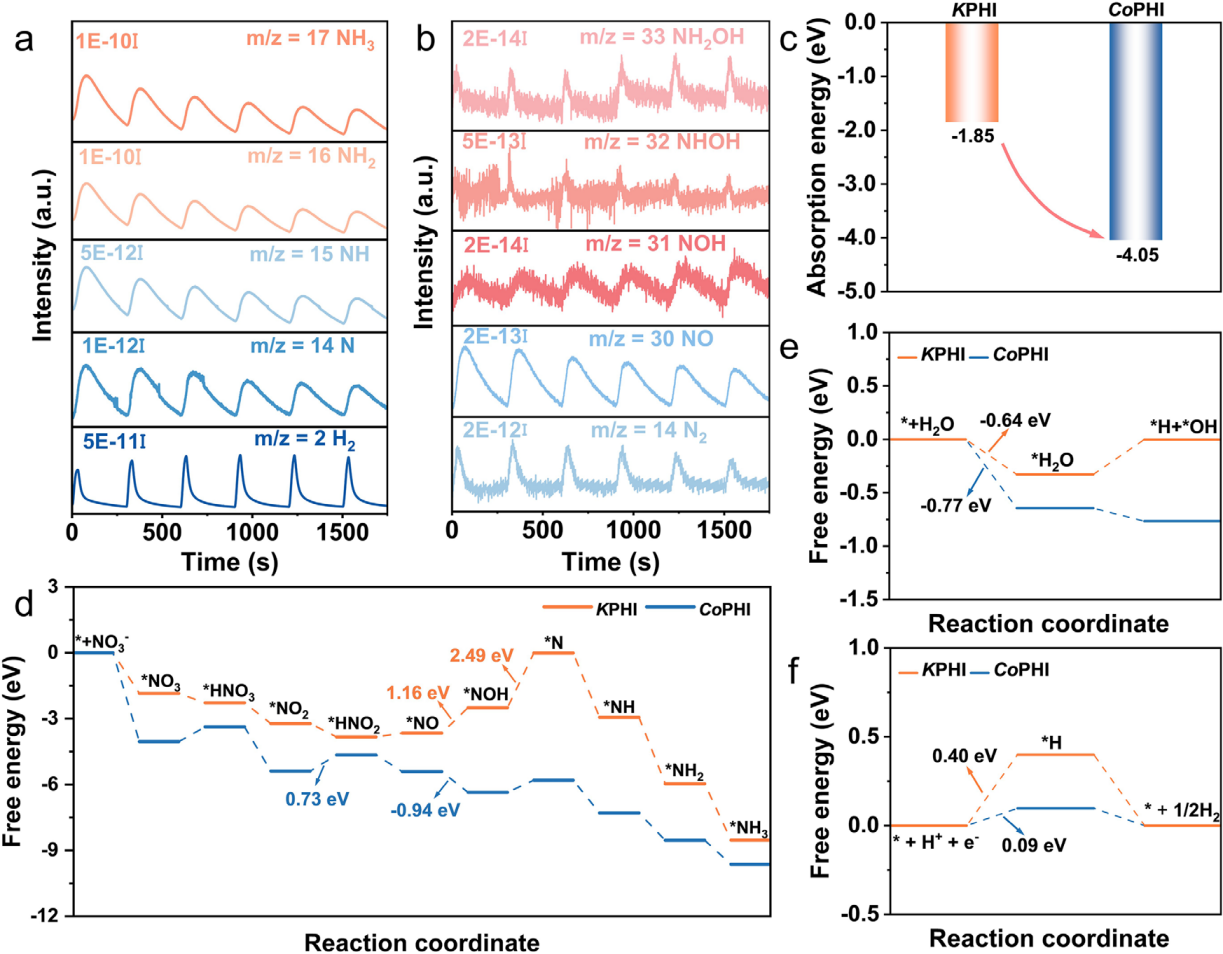
Mechanistic analysis. (a and b) In situ DEMS patterns of *Co*PHI for NO_3_RR. (c) Calculated NO_3_^−^ adsorption energy on the *K*PHI and *Co*PHI models. (d) Free energy diagram of complete NO_3_^−^ to NH_3_ conversion on *K*PHI and *Co*PHI. (e) The calculated free energy in water‐splitting reaction on *K*PHI and *Co*PHI, respectively. (f) Free energy diagram of HER over *K*PHI and *Co*PHI.

To gain deeper insight into the reaction mechanism, we employed in situ differential electrochemical mass spectrometry (DEMS) to identify potential intermediates and products involved in the NO_3_RR. DEMS enables the detection of gaseous species through their characteristic mass‐to‐charge (*m/z*) ratios, providing real‐time information on the relative abundance of reaction species based on signal intensities. During five consecutive constant‐potential cycles at −0.8 V versus RHE, we observed *m/z* signals at 2, 14, 15, 16, 17, 28, 30, 31, 32, and 33, which correspond to H_2_, N, NH, NH_2_, NH_3_, N_2_, NO, NOH, NHOH, and NH_2_OH, respectively (Figure 4a,b). Notably, the signal intensity for NH_3_ was significantly higher than that of N_2_, highlighting the material's excellent catalytic selectivity toward NH_3_ production. The presence of H_2_ (*m/z* = 2) indicated that the HER, a major competing process in NO_3_RR, does occur. However, its signal intensity was approximately one order of magnitude lower than that of NH_3_, confirming that HER plays only a minor role under these conditions (Figure 4a). According to previous studies, NO_3_RR typically proceeds via two primary pathways: (1) *NO_3_ → *NO_2_ → *NO → *NOH → *NH_2_OH → *NH_2_ → *NH_3_ [50, 51, 52, 53]; (2) *NO_3_ → *NO_2_ → *NO → *NOH → *N → *NH → *NH_2_ → *NH_3_ [50, 54, 55, 56, 57]. For *Co*PHI, the DEMS analysis revealed a weak signal corresponding to *NH_2_OH and a strong signal for *N, suggesting that the reduction proceeds predominantly through the second pathway. Along this reaction route, *NO and *N serve as key N‐containing intermediates toward NH_3_ production.

We conducted density functional theory (DFT) calculations to gain insight into the reaction mechanism (Figures S32 and S33). As shown in Figure 4c, the adsorption energy of NO_3_^−^ on *Co*PHI (−4.05 eV) is lower than that on *K*PHI (−1.85 eV), indicating that *Co*PHI has a more favorable NO_3_^−^ adsorption capacity [58]. The Gibbs free energy profiles (Δ*G*) for each reaction step are illustrated in Figure 4d. Both K and Co active sites display a generally descending trend in free energy, indicating that the adsorption‐driven reduction steps are thermodynamically favorable. Initially, NO_3_^−^ adsorbs onto either the K or Co sites, forming the intermediate *NO_3_. This is followed by a protonation step in solution to yield HNO_3_. The subsequent transformation proceeds through eight coupled proton‐electron transfers. However, the energy profiles for the K and Co sites diverge in later steps. The largest free energy barrier, representing the rate‐determining step (RDS), is 2.49 eV for the K site and occurs during the transformation from *NOH → *N. In contrast, the Co site exhibits its RDS during the *NO_2_ → *HNO_2_ step, which requires only 0.73 eV, indicating a significantly more favorable pathway. Among the key steps for NH_3_ formation, the *NO to *NOH transformation is especially critical for some catalysts [59]. For the K site, this step is energetically uphill (+1.16 eV), whereas on the Co site it is thermodynamically downhill (−0.94 eV), implying the superior catalytic potential of Co centers in facilitating NO_3_RR. We further calculated the water splitting process on the two catalysts and found that the water adsorption energy (−0.64 eV) and water dissociation energy (−0.77 eV) on the *Co*PHI surface are lower (Figures 4e and S34), indicating that *Co*PHI can effectively promote water splitting [60, 61]. Moreover, compared to *K*PHI, the relatively low adsorption energy of *H (0.09 eV) on *Co*PHI indicates an accelerated accumulation of *H on the surface, as shown in Figures 4f and S35 [62].

## 3 Conclusions

In summary, we have developed a straightforward strategy to construct Co single‐atom sites on an ionic poly(heptazine imides) framework (*Co*PHI). Benefiting from the negatively charged imide‐bridging nitrogen atoms in PHI, cobalt species are stabilized as highly dispersed atomic centers (> 1 wt.%), while the ionic structure simultaneously enhances charge transport. As a result, *Co*PHI delivers outstanding nitrate‐to‐ammonia electrocatalytic performance, achieving NH_3_ yield rate of 46.1 mg·h^−1^·mg_cat._^−1^, with a FE of 93.5% at −0.8 V versus RHE. This performance significantly exceeds that of analogous *Co–*C_3_N_4_, which only exhibits an NH_3_ yield rate of 967 mg h^−1^ mg_Co_^−1^ with a FE of 52.3% at −0.8 V versus RHE. DFT calculations further indicate that *Co*PHI facilitates the adsorption of nitrate ions on its surface, promotes water dissociation, and enhances the adsorption of *H, thereby favoring the conversion of *NO to *NOH. These findings highlight ionic CNs as a powerful support platform for designing high‐loading single‐atom electrocatalysts for selective nitrate reduction.

## Conflicts of Interest

The authors declare no conflicts of interest.

## Supporting information




**Supporting File 1**: anie71737‐sup‐0001‐SuppMat.docx.

## Data Availability

The data that support the findings of this study are available from the corresponding author upon reasonable request.
