# Supplementary material for: Atomically Dispersed Cobalt on Ionic Carbon Nitrides for Selective and Efficient Nitrate Electroreduction to Ammonia
Source: Angew Chem Int Ed Engl. 2026 Mar 9;65(16):e2543286. doi: 10.1002/anie.2543286 (PMC13080417; doi:10.1002/anie.2543286)
Supplement: Supplementary file 1 — Supporting File 1: anie71737‐sup‐0001‐SuppMat.docx. [file ANIE-65-e2543286-s001.docx]

Supporting Information
©Wiley-VCH 2026
69451 Weinheim, Germany

Atomically Dispersed Cobalt on Ionic Carbon Nitrides for Selective and Efficient Nitrate Electroreduction to Ammonia

Nana Gao+, Minjuan Guo+, Haijian Tong+, Guoyu Hou, Jingwen Ba, Xiaoyu Zhang, Ruixin Zhang, Hui Zhang, Xianwei Fu, Leonardo Cancellara, Nadezda V. Tarakina, Yu Zhang*, Tianxi Liu, Christian Mark Pelicano*, and Zhihong Tian*

**Experimental section**

***Materials and Chemicals.***

KCl (99.5%), LiCl (98%), sodium citrate (99%), NaOH (96%), KNO_3_ (99%), KOH (85%), NaNO_2_ (99%), (NH_4_)_2_SO_4_ (99%), salicylic acid (99.5%), CoCl_2_·6H_2_O (99.8%) and sodium hypochlorite solution (NaClO, ≥5%) were purchased from Tianjin Kemiou Chemical Reagent Company. 5-Aminotetrazole (98%), N-(1-naphthyl) ethylenediamine dihydrochloride (98%), melamine (98%), sodium nitroferricyanide dihydrate (99.98%) and Nafion 117 solution (5 wt.%) were purchased from Shanghai Aladdin Reagent Co., Ltd. Carbon paper (CP, SGL 22BB) and Nafion 117 membrane (Dupont) were purchased from the Gaoss Union (Tianjin) Photoelectric Co., Ltd. Maleic acid (99%), Dimethyl sulfoxide-d_6_ (99.9%) and K^15^NO_3_ (99 atom% ^15^N, purity>99.0%) were purchased from Macklin. Sulfanilamide (99.5%), ethanol (95%) and phosphoric acid (85%) were purchased from Sinopharm Chemical Reagent Co., Ltd. All reagents were used without further purification.

***Synthesis Procedures.***

2.5 g of 5-aminotetrazole, 12.5 g of KCl/LiCl eutectic (0.55/0.45 ratio) and an appropriate amount of CoCl_2_·6H_2_O (nominal metal content is 0.1 wt.% vs. salt melt: 0.0125 g) were placed in a steel ball mill vessel and ground at an operational frequency of 25 Hz for 5 minutes. The resultant white powder was then transferred into a covered porcelain crucible and heated in a furnace. The furnace temperature was gradually increased to 600 °C over 4 hours under a continuous flow of N_2_ gas (5 L min^−1^), and this temperature was maintained for an additional 4 hours. The furnace was subsequently allowed to cool naturally to room temperature. The contents of the crucible were then moved to a beaker containing 250 mL of distilled water and stirred at room temperature overnight. Following this, the product was vacuum filtered, thoroughly washed with water by centrifugation (5 times with 2 mL each, at 13,500 r min^−1^ for 3 minutes), and finally dried in a vacuum oven at 60 °C for 3 hours. The obtained product was designated as *Co*PHI. *Co*PHI samples with different cobalt content were prepared by adding 0.05 wt.% and 2 wt.% of CoCl_2_·6H_2_O, respectively, to the KCl-LiCl salt template while keeping all other conditions constant. The same procedure was repeated without adding cobalt salt while keeping all other steps unaltered, yielding the *K*PHI sample. A mixture of 2.5 g melamine and 0.06 g cobalt chloride was placed in a ball milling container and ground according to the aforementioned ball milling parameters. The resulting powder was then transferred to a covered porcelain crucible and placed in a muffle furnace. N_2_ gas was introduced for 1 hour to purge air from the system. Subsequently, under a continuous N_2_ gas (1 L·min^-1^), the temperature was raised to 550 °C at a heating rate of 2.3 °C·min^-1^ and maintained for 4 hours. After cooling naturally, the product was thoroughly washed with water and finally dried in a vacuum oven at 60 ℃. The obtained product was designated as *Co*-C_3_N_4_. All other steps remained unchanged. Without the addition of cobalt salt, the pyrolysis was carried out according to the aforementioned method to obtain the C_3_N_4_ sample.

***Characterizations.***

Scanning electron microscopy (SEM) images were captured using Gemini SEM 500. X-ray diffraction (XRD) patterns were collected on Bruker D8 Phaser using Cu Kα radiation. Transmission electron microscopy (TEM) images and scanning transmission electron microscopy (STEM) images, energy dispersive X-ray spectroscopy (EDS) elemental mapping and high-resolution transmission electron microscopy (HRTEM) images were acquired with Japan JEOL-JEM 2100 F TEM. Aberration corrected high-angle annular dark-field scanning transmission electron microscopy (AC HAADF-STEM) images were obtained using a JEOL JEMARM200F operated at 200 kV, equipped with a spherical aberration correction system. Inductively coupled plasma optical emission spectrometer (ICP-OES) analyses were conducted with ICP-OES Agilent 5110, and inductively coupled plasma-mass spectrometry (ICP-MS) analysis was conducted on an Agilent-7850 ICP-MS. X-ray photoelectron spectroscopy (XPS) measurements were performed on Thermo SCIENTIFIC ESCALAB 250Xi. All peaks were calibrated against the C 1s spectrum at a binding energy of 284.8 eV. UV–vis spectra were recorded on ultraviolet spectrophotometer (UV-2600, Japan Shimadzu). ^15^NH_4_^+^ in the reaction solution was conducted with ^1^H nuclear magnetic resonance (NMR) measurements (500 MHz) at room temperature. Fourier transform infrared (FT-IR) spectroscopy was measured on a Bruker Tensor Ⅱ FTIR spectrometer in the attenuated total reflection (ATR) mode with 64 scans and resolution of 4 cm^-1^. The electronic structure and local coordination environment of Co atoms of the sample were determined by X-ray absorption near edge structure (XANES) and Extended X-ray absorption fine structure (EXAFS) on the NFPS BL17B1 of Shanghai Synchrotron Radiation Facility (SSRF). This beamline adopted fixed-exit double crystal Si (111) monochromator to ranging the X-ray energy from 5-23 keV. The end-station equipped three ionization chambers and Lytle detector for transmission and fluorescence mode X-ray absorption spectroscopy. The beam size of X-ray on the sample is about 1.5×0.6 mm (H×V), and the maximum luminous flux is 2.2×10^11 phs/s @12 keV.

***Electrochemical Measurements.***

The electrochemical measurements were performed on the traditional three-electrode system in a typical H-cell separated by a Nafion 117 member, with Hg/HgO as the reference electrode and platinum mesh as the counter electrode. To prepare the working electrode, 2 mg of the as-prepared sample was dispersed into the mixture of 0.19 mL water, 0.05 mL ethanol, and 0.01 mL Nafion 117 solution, under ultrasonication for 30 min. Subsequently, 0.05 mL catalyst solution was dropped on the 1 × 2 cm^2^ carbon paper to form the 1 × 1 cm^2^ catalyst electrode. KOH (0.1 M) was used as an electrolyte added into the anode and cathode compartments, and 1.0 M KNO_3_ was also contained in the anode and cathode compartments. Before the electrochemical measurement, argon was bubbled into the cathode electrolyte for 20 min to exclude the nitrogen interference and kept bubbling under the measurement process. All potentials obtained in the measurements were converted to the values versus reversible hydrogen electrode (RHE) by the following equation:

E_RHE_ = E_Hg∕HgO_ + 0.098 + 0.059 × pH (1)

Determination of ECSA: The electrochemically active surface area (ECSA) was compared by comparing the samples’ electrochemical double-layer capacitance. The double-layer capacitance value was extracted from the CV curves measured in non-faradic region of the samples based on the following equation:

*C_dl_ = I_c_/v* (2)

Where *Ic* is the charging current density (mA cm^-2^), and *v* is scan rate (mV s^-1^). In our case, the scan rates of 20, 40, 60, 80, 100 mV s^-1^ were applied.

***Determination of Electrochemical Products.***

Determination of produced NH_4_^+^: The concentration of NH_4_^+^ was determined by the indophenol blue method. Two milliliters of mixed solution (1.0 M NaOH, 5 wt.% salicylic acid, and 5 wt.% sodium citrate), 1.0 mL NaClO (0.05 M), and 0.2 mL sodium nitroferricyanide dihydrate (1 wt.%) were added into the 2.0 mL diluted electrolyte solution, followed by the measurement on the absorption intensity at wavelength of 655 nm after 0.5 h incubation.

Determination of produced NO_2_^-^: The concentration of NO_2_^-^ was determined by the typical Griess method. Griess solution (1.0 mL) (1.0 g sulfanilamide, 0.1 g N-(1-naphthyl) ethylenediamine dihydrochloride, 2.94 mL H_3_PO_4_, and 50.0 mL water) and 2.0 mL water were added into the 1.0 mL diluted electrolyte solution, followed by the measurement on the absorption intensity at a wavelength of 540 nm after 10 min incubation.

The yield rate of the product was calculated by the following equation:

Yield rate = $\frac{c \times V}{t \times m}$ (3)

Where *c* is the concentration of the product, *V* is the volume of the electrolyte in the cathode compartment, *t* is the electrolysis time, and *m* is the quality of the catalyst.

The FE was calculated by the following equation:

FE = $\frac{n \times F \times c \times V}{M\times Q}$ (4)

Where *n* is the number of electrons transferred during the formation of 1.0 mol product, *F* is the Faradaic constant (96485 C mol^-1^), *c* is the concentration of the product, *V* is the volume of the electrolyte in the cathode compartment, *M* is the molar mass of product and *Q* is the total charge consumed in the electrolysis process.

Determination of NH_4_^+^ using ^1^H nuclear magnetic resonance (NMR, 500 MHz) spectroscopy: The concentration of NH_4_^+^ was quantitatively determined by ^1^H NMR, using DMSO-d_6_ as a solvent and maleic acid (C_4_H_4_O_4_) as the internal standard solution. To prepare the calibration curve, a series of NH_4_Cl solutions with known concentrations was prepared. Then, 0.5 mL of standard solution was mixed with 0.1 mL of internal standard solution (containing 0.04 wt.% C_4_H_4_O_4_; 20 mg of C_4_H_4_O_4_ in 50 g of DMSO-d_6_). Subsequently, the mixture was examined with a ^1^H NMR spectrometer at room temperature. Finally, the calibration curve was obtained by using the ^1^H peak area ratio of NH_4_^+^/C_4_H_4_O_4_ and the known concentration of NH_4_^+^. The obtained calibration equation was used to determine the concentration of NH_4_^+^ after NO_3_^-^ reduction. The pH of the electrolyte was adjusted to 2.0 using 1.0 M HCl. Then, the NH_4_^+^ in the electrolyte was detected using the same method as for the calibration curve. The amount of NH_4_^+^ generated can be calculated from the peak area ratio using the calibration curve.

^15^N isotope-labeling experiment: Isotope labeling experiment was conducted to determine the source of NH_3_, using a mixed solution of 0.1 M KOH and 0.1 M K^15^NO_3_ (99 atom% ^15^N) as the electrolyte. ^15^NH_4_^+^ was detected by ^1^H NMR following the electroreduction of ^15^NO_3_^-^ for 1 h at a potential of -0.8 V vs. RHE. The NMR testing method for ^15^NH_4_^+^ is identical to that of ^14^NH_4_^+^.

***The Calculation of the Turnover frequency (TOF)*.**

TOF = $\frac{n}{N\times t}$ (5)

Where, n denotes molar amount of the product, N is the molar quantity of active metal sites as determined by ICP-AES analysis, t represents the electrolysis time (h).^[1]^

***In situ DEMS measurement.***

For DEMS testing, the electrolyte containing 1.0 M KNO_3_ in 0.1 M KOH was continuously pumped into a dedicated electrochemical cell using a peristaltic pump. Ar gas was continuously introduced before and during the test. Using carbon paper loaded with catalyst, a platinum wire, and a saturated Ag/AgCl electrode as the working electrode, counter electrode, and reference electrode respectively, constant potential testing was conducted at -0.8 V vs. RHE, and a corresponding mass signal was observed. After the electrochemical test, the mass signal returned to the baseline, and the next cycle was initiated. The experiment concluded after six cycles.

***Calculation details.***

All calculations based on density functional theory (DFT) were performed using the Perdew-Burke-Ernzerhof (PBE) functional within the generalized gradient approximation (GGA) framework, implemented with the Vienna ab initio simulation package (VASP). A plane-wave energy cutoff of 500 eV was employed in the calculations, and a 3×3×1 k-point mesh generated by the Monkhorst-Pack scheme was used for sampling the Brillouin zone integration in reciprocal space. In the geometric optimization process, the energy convergence threshold of 10^-5^ eV and the force convergence threshold of 0.02 eV/Å are adopted, respectively. The Gibbs free energy (ΔG) for each elementary step in the NO_3_RR process can be calculated based on the computational hydrogen electrode (CHE) method, as described below:

ΔG = ΔE_DFT_ + ΔE_ZPE_ – TΔS (6)

Where, ΔE_DFT_, ΔE_ZPE_, and ΔS are the energy computed by density functional theory, the changes of zero-point energy (ZPE), and the entropy change, respectively.

**Figures** **and** **Tables**

**Table S1.** Metal loading measured by ICP-OES for the catalysts.

| **Material** | **Li (m/m %)** | **K (m/m %)** | **Co (m/m %)** |
| --- | --- | --- | --- |
| *Co*PHI | 0.92 | 9.19 | 1.092 |
| *K*PHI | 0.98 | 9.34 | / |
| *Co*-C_3_N_4_ | / | / | 0.94 |
| *Co*PHI (After 4 h) | 0.77 | 12.01 | 1.088 |
| Protonated *Co*PHI | 0.10 | 0.14 | 0.14 |

**Table S2.** Curve fit parameters of Co K-edge EXAFS for the *Co*PHI.

| **Sample** | **Path** | **N** | **R (Å)** | **σ^2^** **(10^-2^ Å^2^)** | **ΔE_0_ (eV)** | ***R* factor** |
| --- | --- | --- | --- | --- | --- | --- |
| *Co*PHI | Co-N | 3.71±0.80 | 2.03±0.018 | 1.35±0.32 | -2.64±2.21 | 0.013 |

Data ranges: 3.0 ≤ k ≤ 9.0 Å^-1^, 1.0 ≤ R ≤ 2.6 Å. N^b^: coordination number; R: interatomic distance; σ^2^: Debye-Waller factor; ΔE_0_: inner potential shift; R-factor: the goodness of fit parameters.

**Table S3.** Comparison of the NO_3_RR of *Co*PHI with recently reported electrocatalysts.

| **Catalysts** | **TOF**  **(h^-1^)** | **FE (%)** | **NH_3_ yield rate**  **(mg h^-1^ mg_cat._^-1^)** | **NH_3_ yield rate (mg h^-1^ mg_metal_^-1^)** | **Ref.** | |  |
| --- | --- | --- | --- | --- | --- | --- | --- |
| *Co*PHI | 13821 | 93.5 | 46.1 | 4221.6 | | This work | |
| Co(OH)_2_@Co-N-C | - | 81.4 | 1.39 | - | | ^[2]^ | |
| Cu/NC | 22969 | 100 | 32.3 | 6460 | | ^[3]^ | |
| Ru SA-NC | 76790 | 70.7 | 51.75 | 16172 | | ^[4]^ | |
| Fe-SAC | 4120.7 | 75 | 20 | 1324.5 | | ^[5]^ | |
| HE Cu_1_-N_4_ | 999.8 | 100 | 5.09 | 281.2 | | ^[6]^ | |
| Fe1/NC-900 | 13290.7 | 86 | 18.8 | 4272 | | ^[7]^ | |
| Ru1-Co/HCO | 2250.7 | ~100 | 11 | 474 | | ^[8]^ | |
| Cu-SAC | 490.7 | 70 | 6.96 | 138 | | ^[9]^ | |
| Co_3_O_4_ | - | > 99 | 5.73 | - | | ^[10]^ | |
| Co_3_O_4_@CNF | - | 92.7 | 23.4 | - | | ^[11]^ | |
| Fe_2_Co-MOF | - | 90.55 | 20.65 | - | | ^[12]^ | |
| CoTiO_3−x_ | - | 92.6 | 30.4 | - | | ^[13]^ | |
| Co1-P/NPG | - | 93.8 | 8.6 | - | | ^[14]^ | |
| Pd@FeNiCoO | - | 69 | 20.26 | - | | ^[15]^ | |
| Co/CoO NSA | - | 93.8 | 3.31 | - | | ^[16]^ | |
| Mg_0.2_Co_0.2_Ni_0.2_Cu_0.2_Zn_0.2_O | - | 93.4 | 4.84 | - | | ^[17]^ | |
| Cu-SNC-2-SAC | 1660 | 98.2 | 4.25 | 466.8 | | ^[18]^ | |
| CuSA/V-LDH | - | 95.2 | 2.08 | - | | ^[19]^ | |
| Cu−N−C SAC | 800 | 84.7 | 2.25 | 225 | | ^[20]^ | |
| Cu–NCOM | 672 | 96.7 | 4.44 | 189 | | ^[21]^ | |
| Cu@Fe1-NC | 125 | 97.7 | 4.72 | 35.17 | | ^[22]^ | |
| Cu-N3 SAC | 251.7 | 89.64 | 30.09 | 70.8 | | ^[23]^ | |
| 1-Cu | 190 | 85.5 | 1.02 | 53.43 | | ^[24]^ | |
| Cu MNC | 349.9 | 94.8 | 0.48 | 98.4 | | ^[25]^ | |
| Co SAAC/INC | 934.7 | 97.2 | 18.64 | 285.5 | | ^[26]^ | |

**
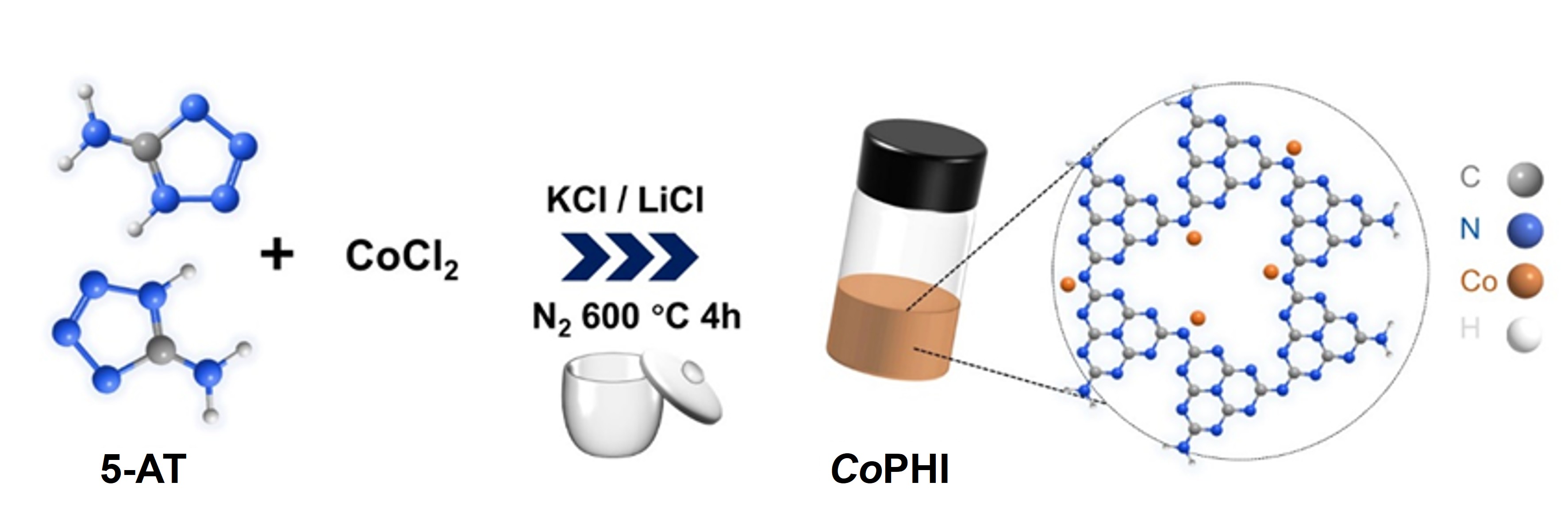
**

**Figure** **S1**. Schematic illustration of *Co*PHI formation (5-AT represents 5-aminotetrazole).


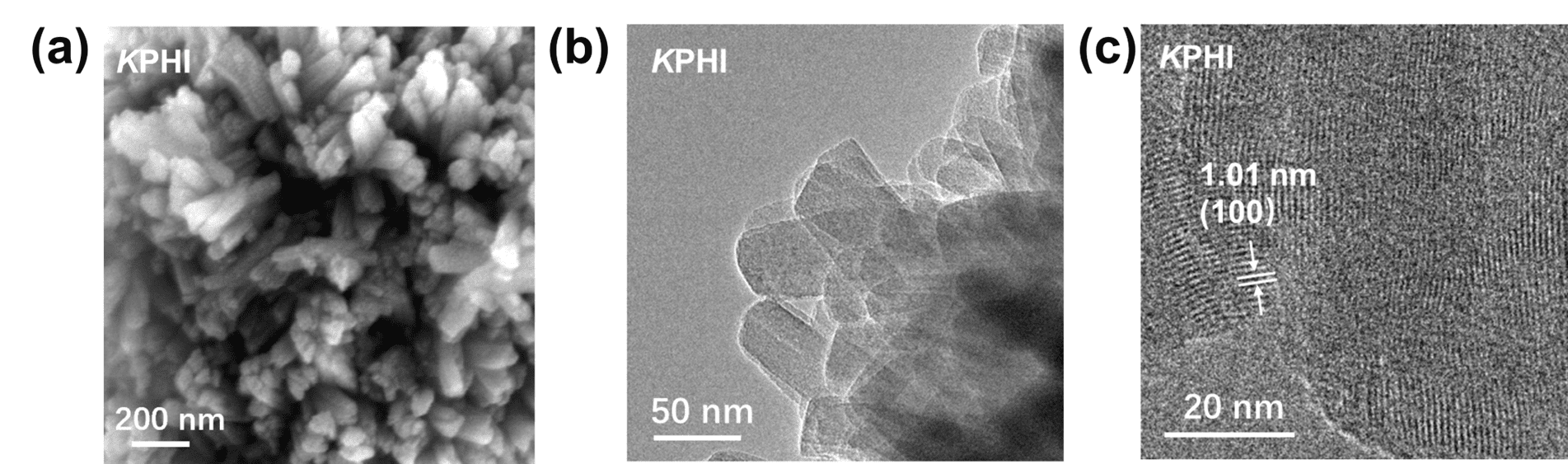


**Figure** **S2.** (a) SEM image, (b) TEM image and (c) HRTEM image of *K*PHI.


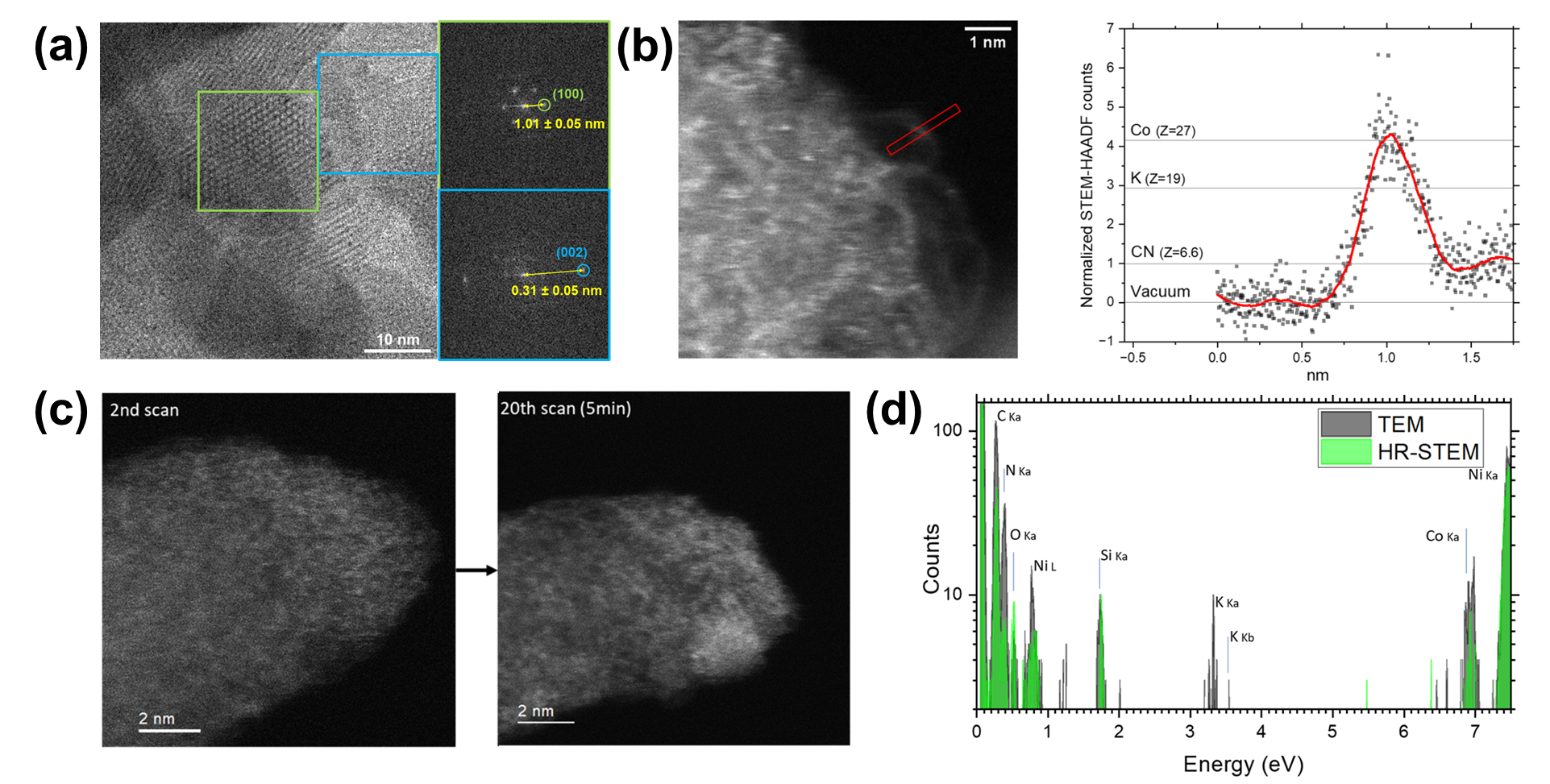


**Figure** **S3.** (a) HRTEM image and the corresponding fast Fourier transform (FFT) patterns of the square regions, (b) AC HAADF-STEM image and its corresponding particle size distribution histogram. (c) HR-STEM images and (d) the corresponding energy dispersive X-ray (EDX) spectra of *Co*PHI.

Observation and identification of different metal ions in graphitic carbon nitride smatrices is challenging due to the extreme sensitivity of these materials to the high energies used in typical STEM observations, as well as the presence of unknown and changing thickness gradients in the sample. Due to damage mechanisms such as radiolysis, knock-on displacement, and heating, the matrix structure degrades significantly within just a few seconds of exposure to the electron probe, which in turn makes weakly coordinated metal ions mobile during image acquisition. Alkali metal ions such as K⁺ are furthermore known to be highly mobile under electron beam irradiation due to induced electric field effects. Mobile K species may therefore contribute to the total HAADF intensity of matrix areas (proportional to the interaction volume's average Z) but cannot be reliably visualized as discrete single atoms with the scan dwell times used for imaging. That the bright dots in Figure 1d are indeed Cobalt (Co) atoms can be assessed by comparing HAADF intensities in the thinnest region, assuming negligible crystallographic contrast: normalizing the vacuum as 0 and the surrounding material as 1, the resulting value of 4.15 matches the Z-dependence expected for Co (Z=27) but not for K (Z=19) (Figure S3b).

The concurrent decrease in both K and N signals during HR-STEM observation provides additional evidence that the stable bright atomic spots originate from Co rather than K. A reference EDX spectrum was first acquired in TEM mode (lower magnification and lower electron dose, higher total counts) for 5 minutes, and subsequently in HR-STEM mode for the same duration. The HR-STEM acquisition resulted in severe deterioration of the CN matrix, mass loss, and progressive clustering of the bright metal atoms. Nitrogen and potassium peaks are particularly weak in the HR-STEM spectrum, indicating that mass loss during degradation arises predominantly from these two species (Figure S3c-d).

The loss of K signal alongside N correlates well with K species being only weakly coordinated to the CN matrix, likely through nitrogen sites that are destroyed during beam-induced degradation. When N is released during degradation, K ions lose their anchoring coordination environment, and they either desorb from the sample surface, migrate to beam-damaged regions outside the field of view, or become mobilized in the degrading carbon framework.

In contrast, despite the damage and extreme sparsity of photon counts, a clear Co Kα peak can be identified in both spectra (i.e., before and after matrix damage), confirming the relative resilience of Co atoms to electron beam exposure. This demonstrates that Co forms much stronger coordination bonds that persist longer under beam irradiation. The differential stability under electron beam exposure (with Co remaining atomically anchored while K is progressively lost) further confirms that the bright isolated spots in Figure 1d correspond to Co single atoms rather than K species. Nickel and Silicon signals arise from the TEM grid and the EDX detector, respectively.


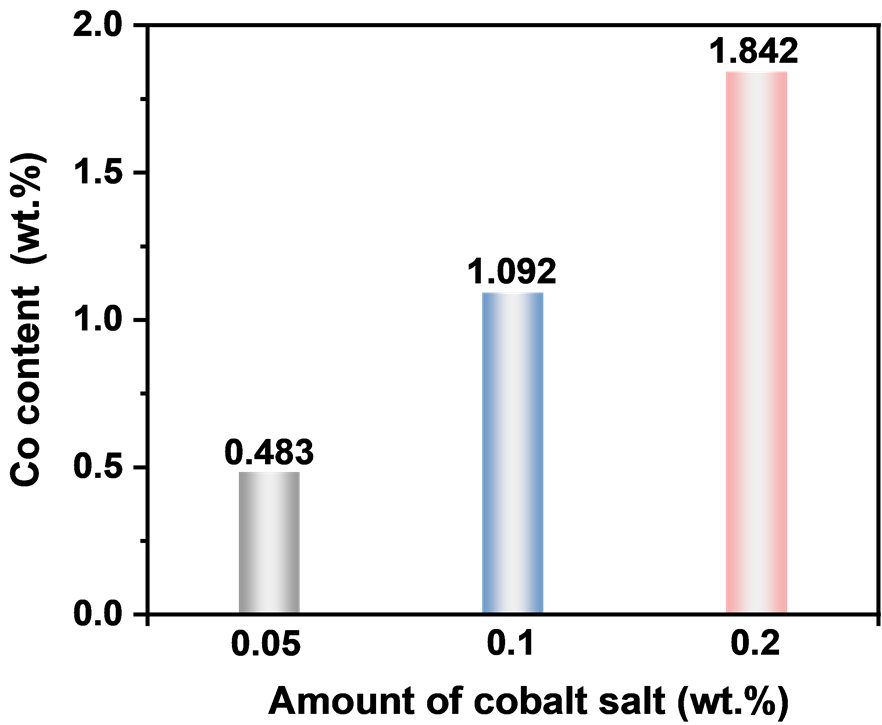


**Figure S4.** Co content measured by ICP-OES for the *Co*PHI with different cobalt contents.


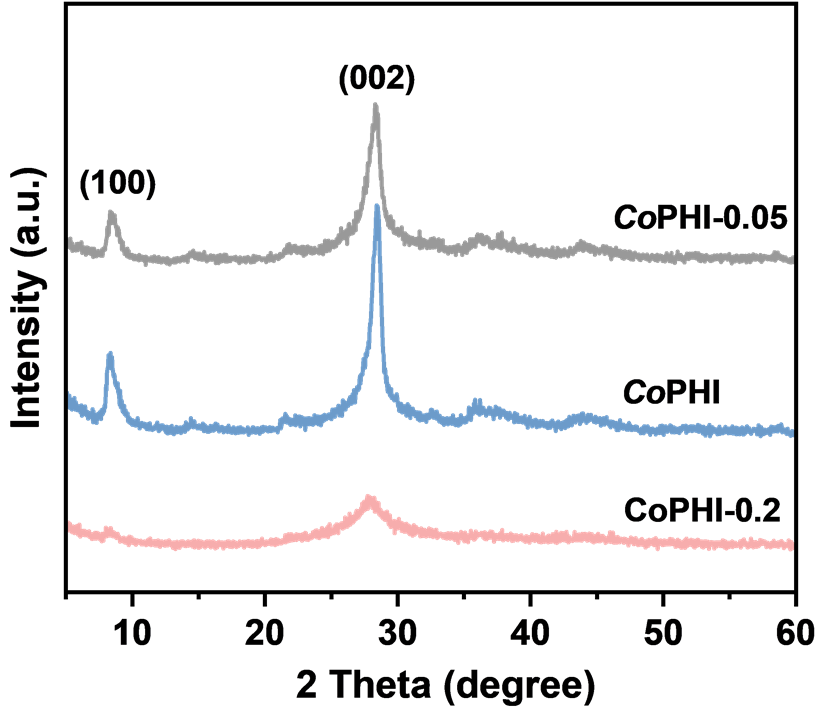


**Figure S5.** XRD patterns of *Co*PHI with different cobalt contents.


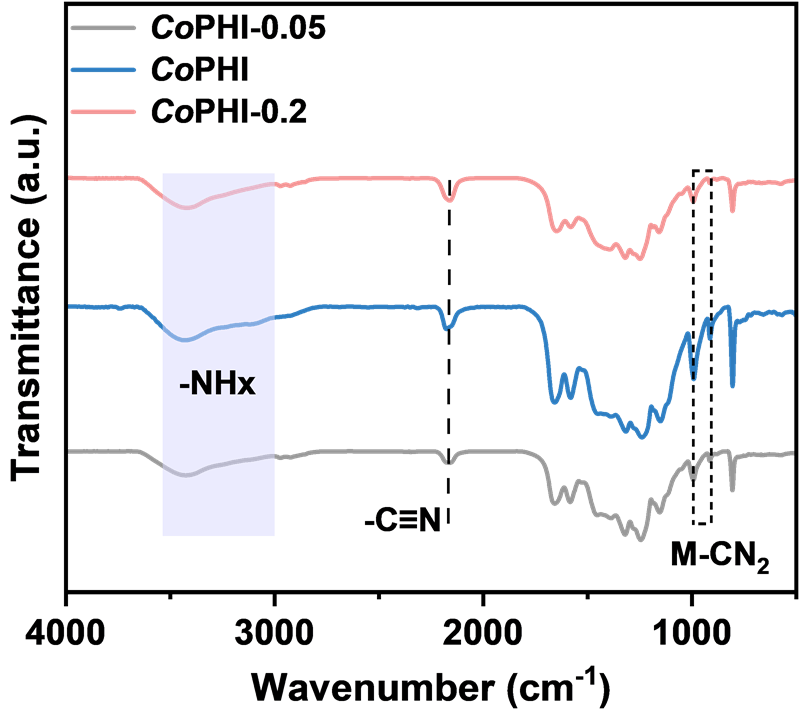


**Figure S6.** FT-IR spectra of *Co*PHI with different cobalt contents.


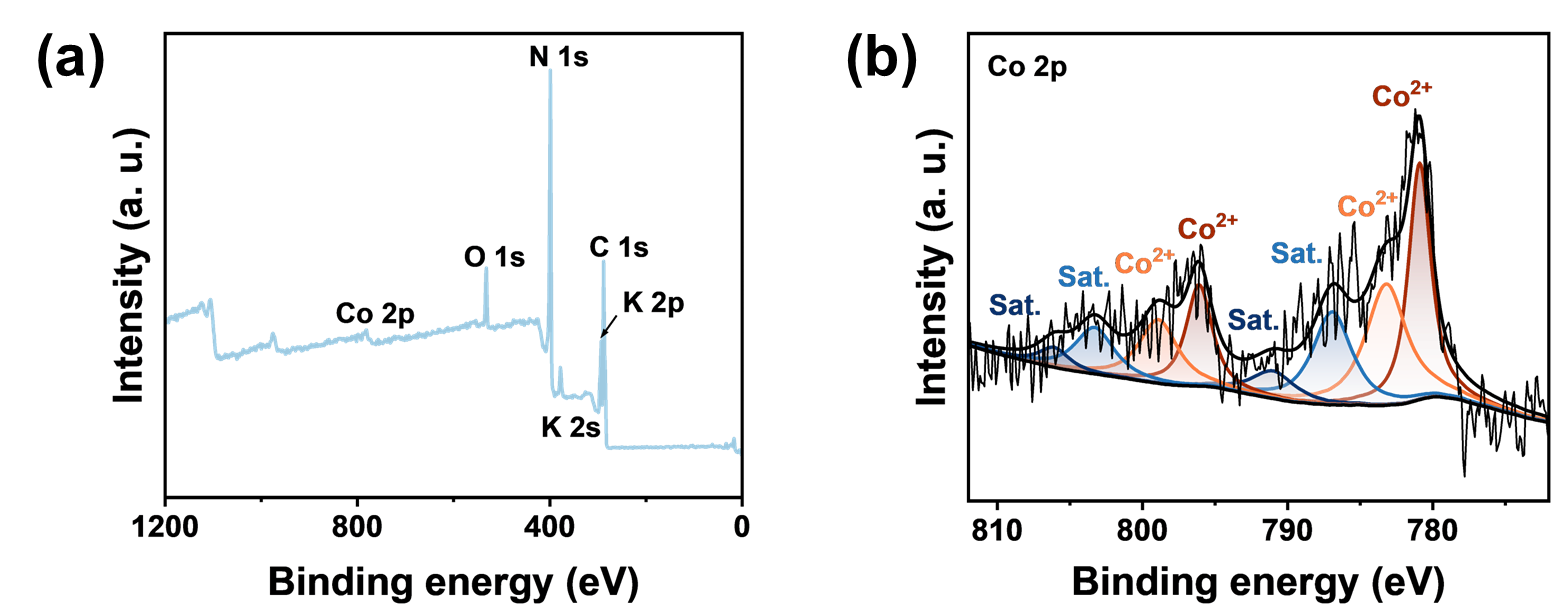


**Figure** **S7.** (a) XPS survey spectrum and (b) high-resolution Co 2p spectrum of *Co*PHI.


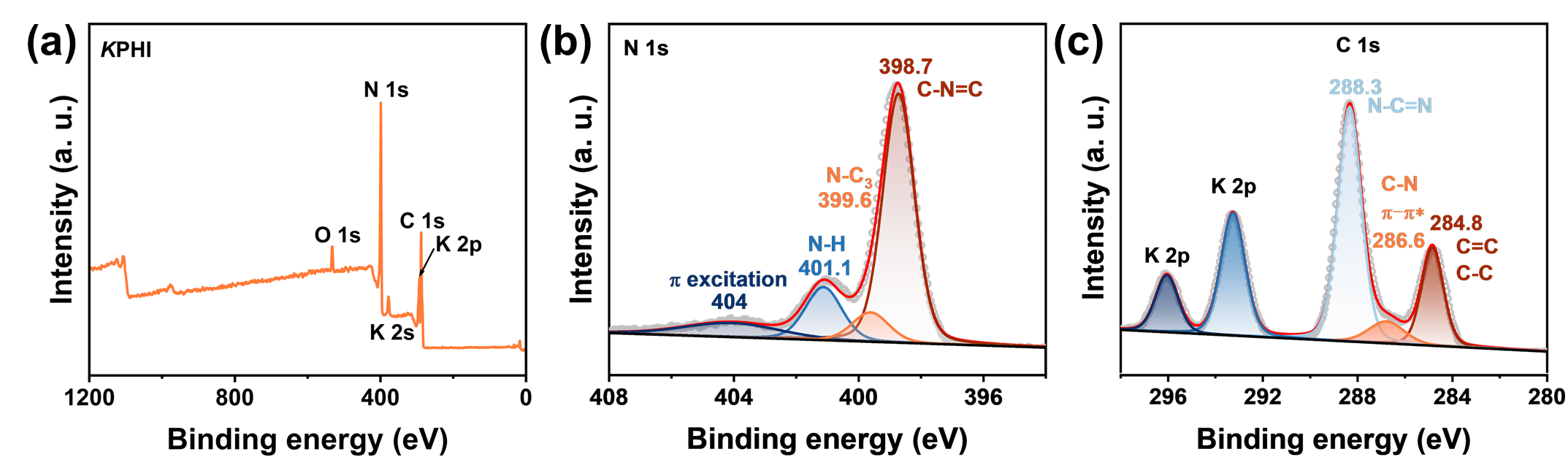


**Figure** **S8.** (a) XPS survey spectrum, (b) high-resolution N 1s and (c) C 1s spectrum of *K*PHI.


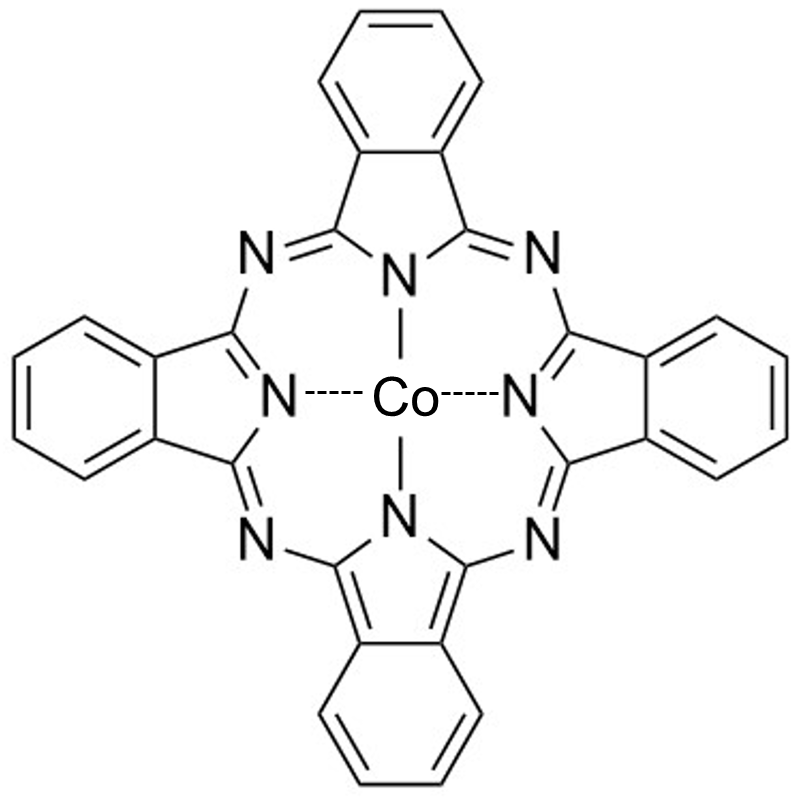


**Figure S9**. The structural formula of cobalt phthalocyanine (CoPc).


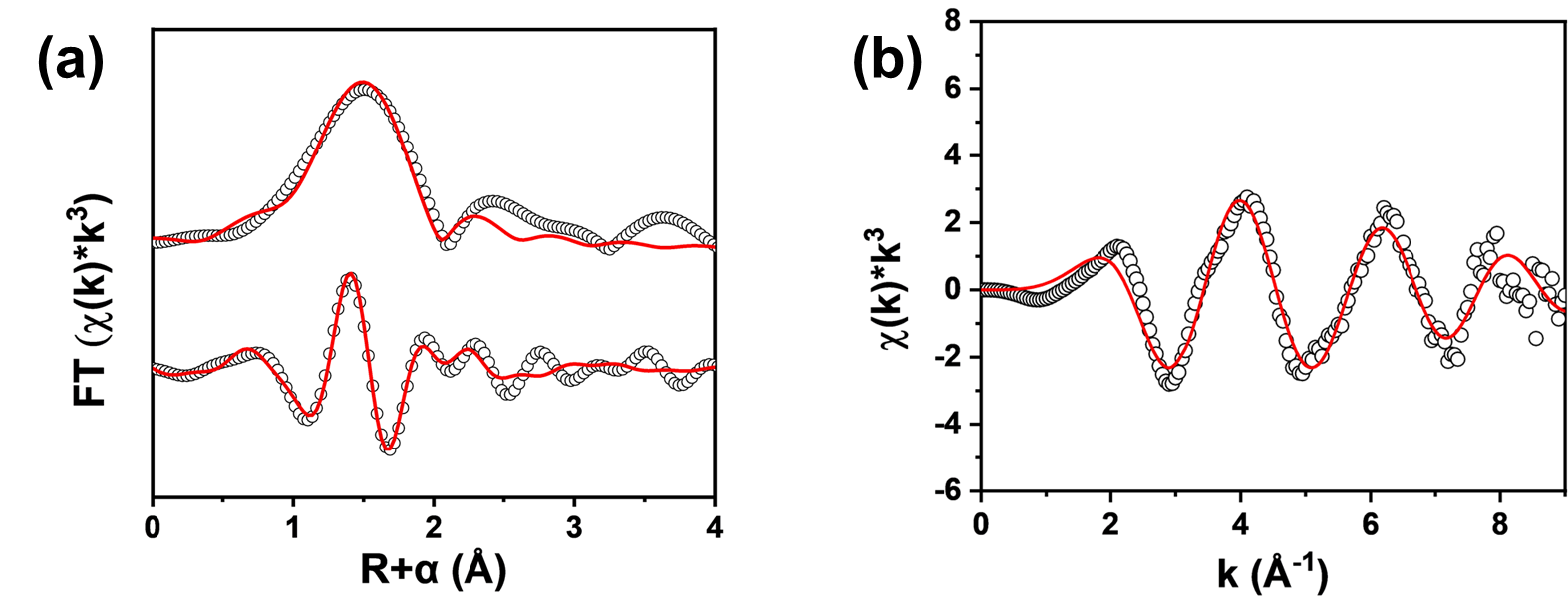


**Figure S10**. (a) R-space and (b) k-space plots of the k3-weighted Co K-edge EXAFS spectrum for *Co*PHI, along with the corresponding fitting curves.


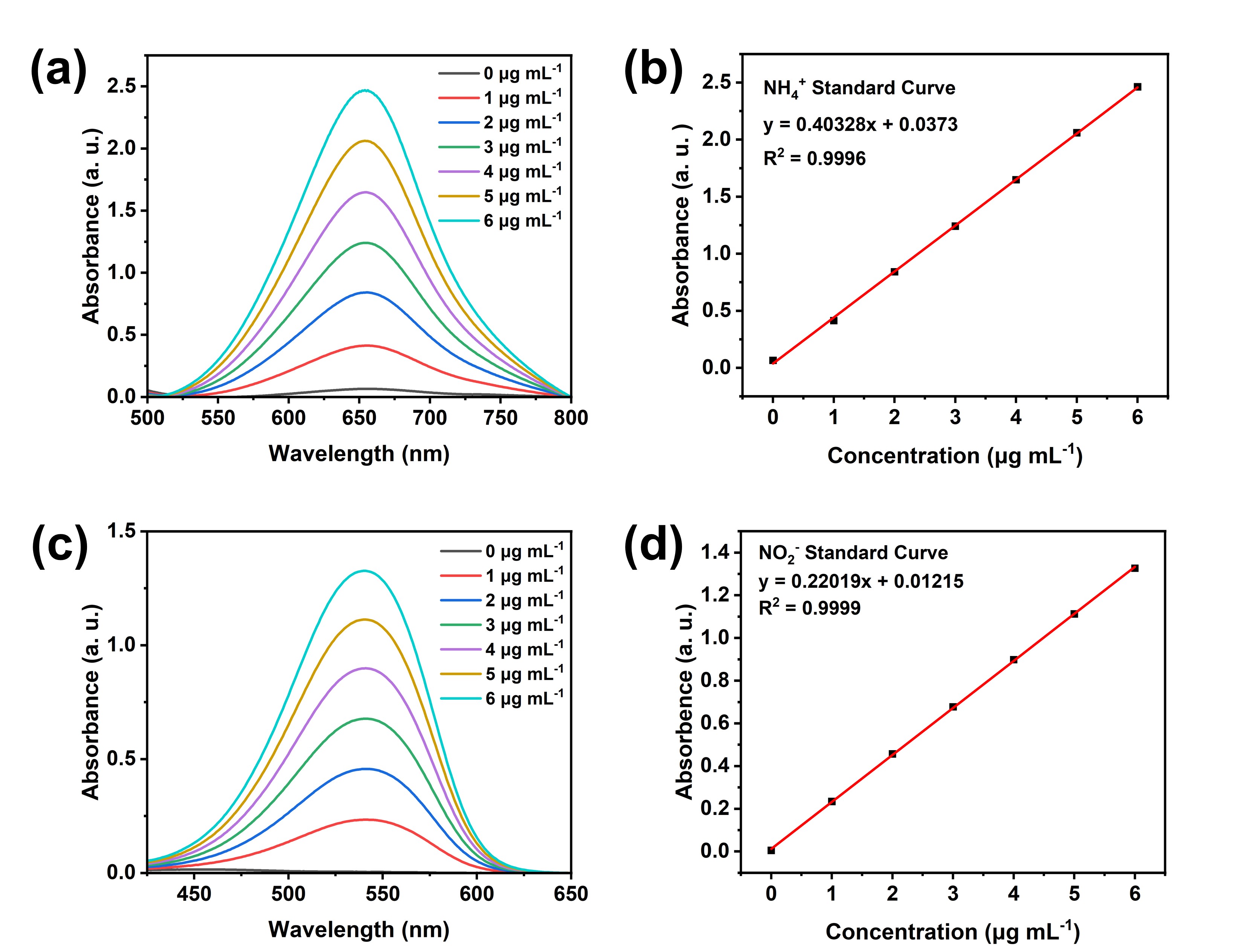


**Figure** **S11.** UV-vis spectra of (a) NH_4_^+^ and (c) NO_2_^-^ with different concentrations. The corresponding linear fitting of the absorbance at (b) 655 nm versus the concentration of NH_4_^+^ and (d) 540 nm versus the concentration of NO_2_^-^.


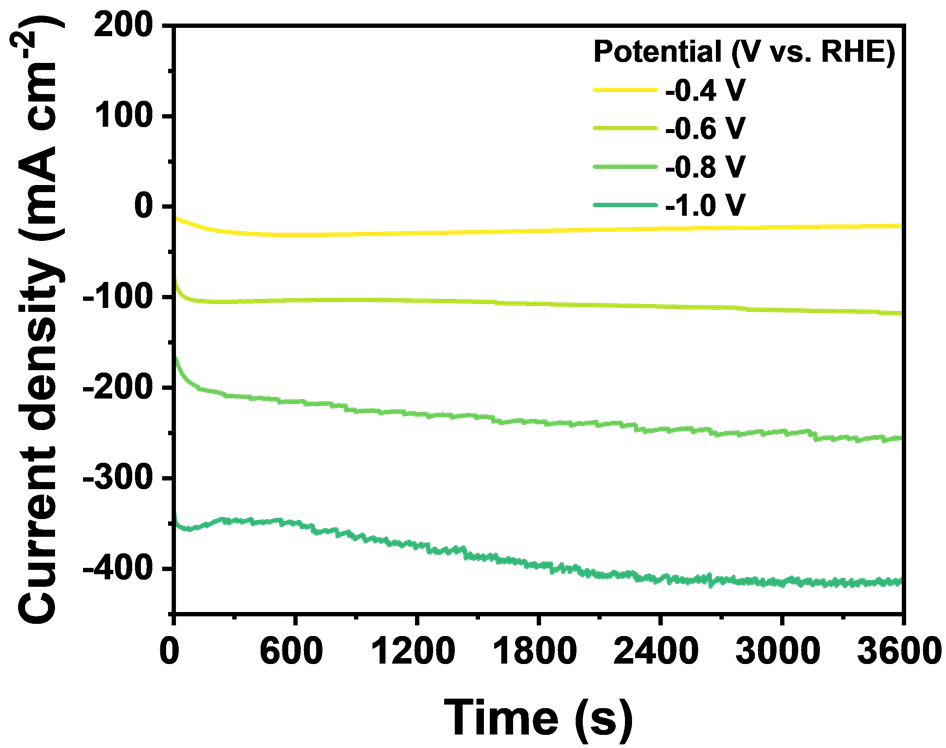


**Figure** **S12.** The current density versus time at different potentials by using *Co*PHI electrode.


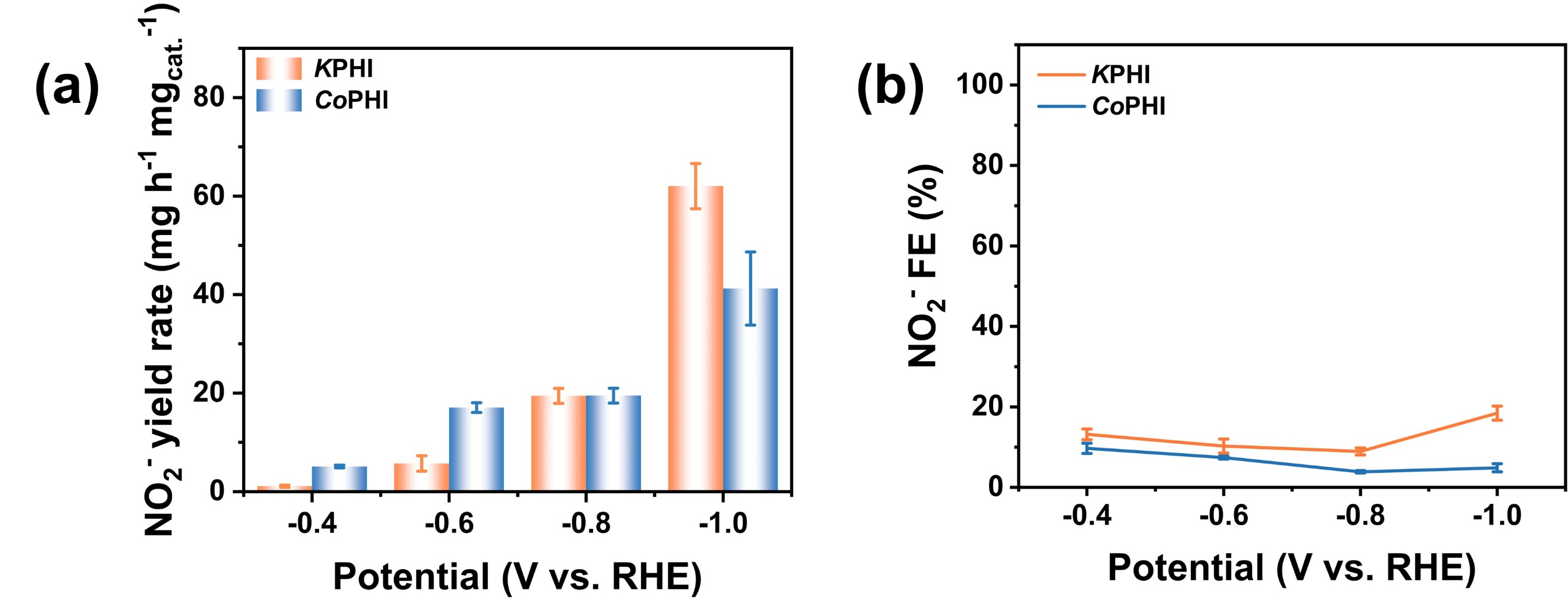


**Figure** **S13.** (a) NO_2_^-^ yield rate and (b) NO_2_^-^ FE of *K*PHI and *Co*PHI at different potentials.


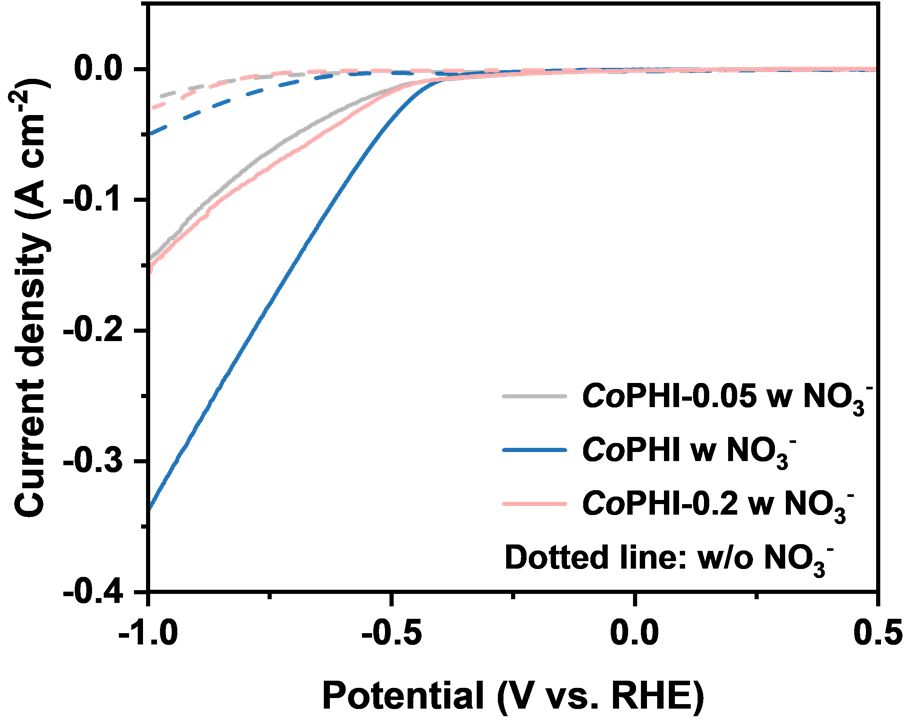


**Figure S14**. LSV curves of *Co*PHI with different cobalt content in 0.1 M KOH with or without 1.0 M KNO_3_.


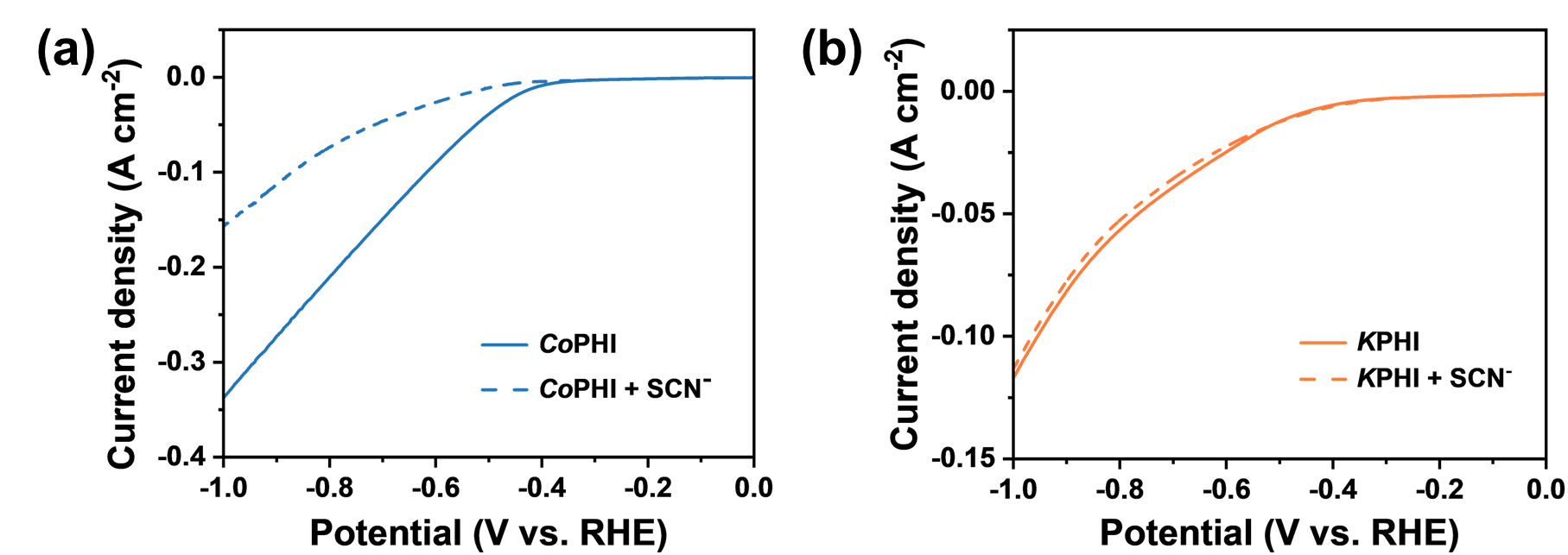


**Figure S15.** LSV curves of (a) *Co*PHI and (b) *K*PHI before and after poisoning with KSCN in 0.1 M KOH with 1.0 M KNO_3_.


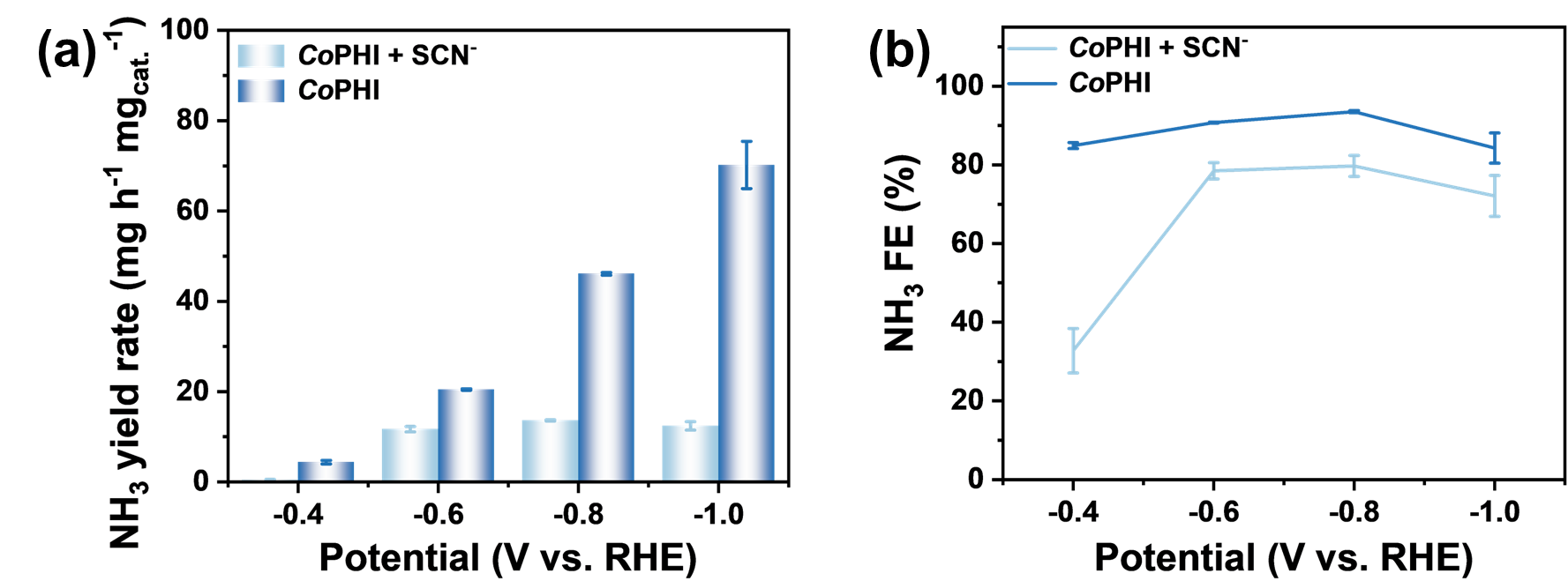


**Figure S16.** (a) NH_3_ yield rate and (b) NH_3_ FE of *Co*PHI at different potentials before and after poisoning with KSCN.


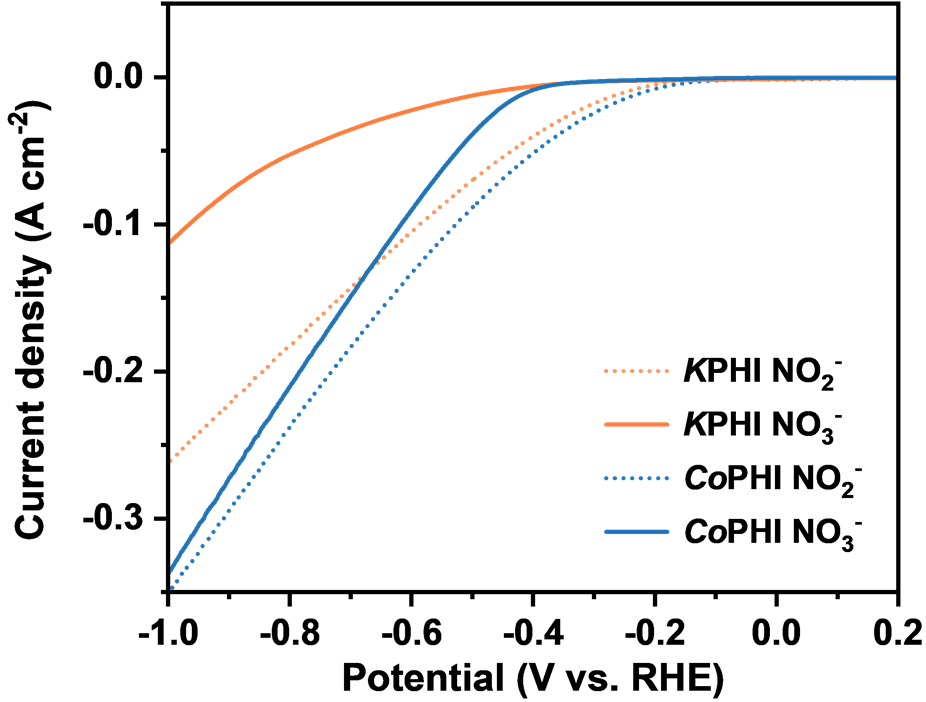


**Figure S17.** Different LSV curves of *K*PHI and *Co*PHI with NO_3_^-^ or NO_2_^-^.


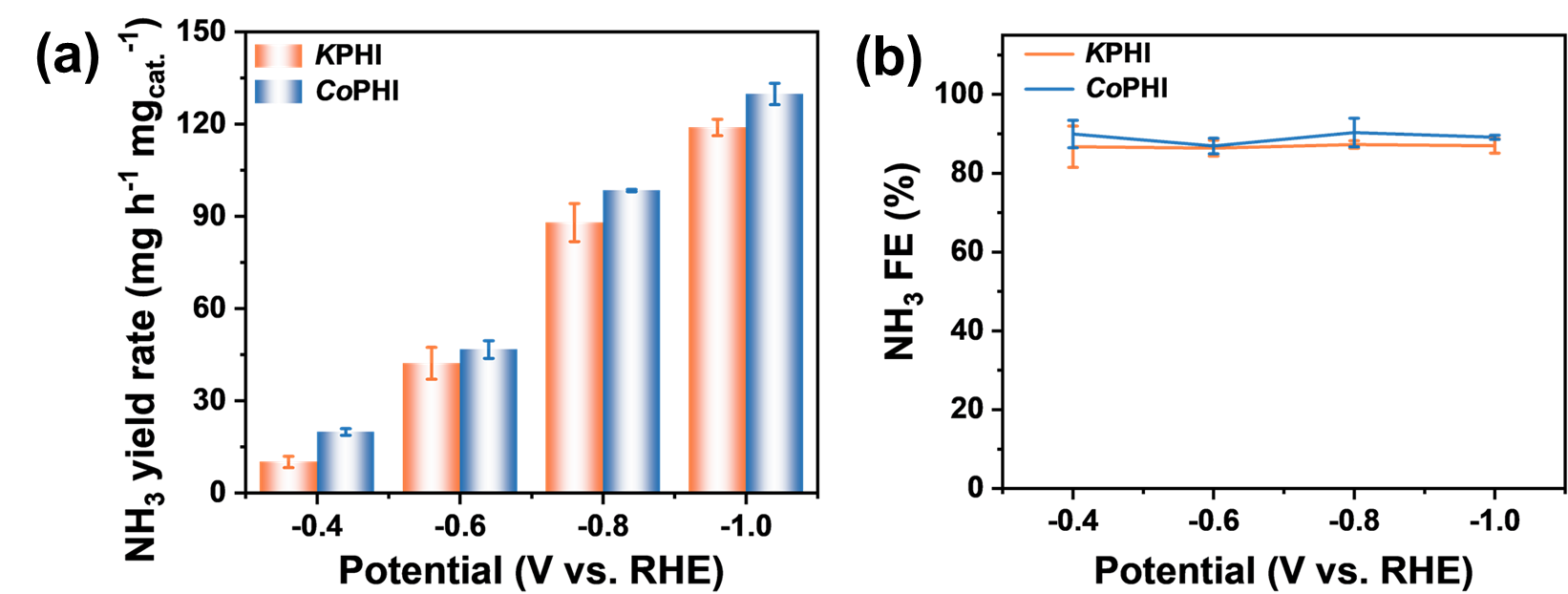


**Figure S18.** (a) NH_3_ yield rate and (b) NH_3_ FE of *K*PHI and *Co*PHI on NO_2_RR at different potentials.


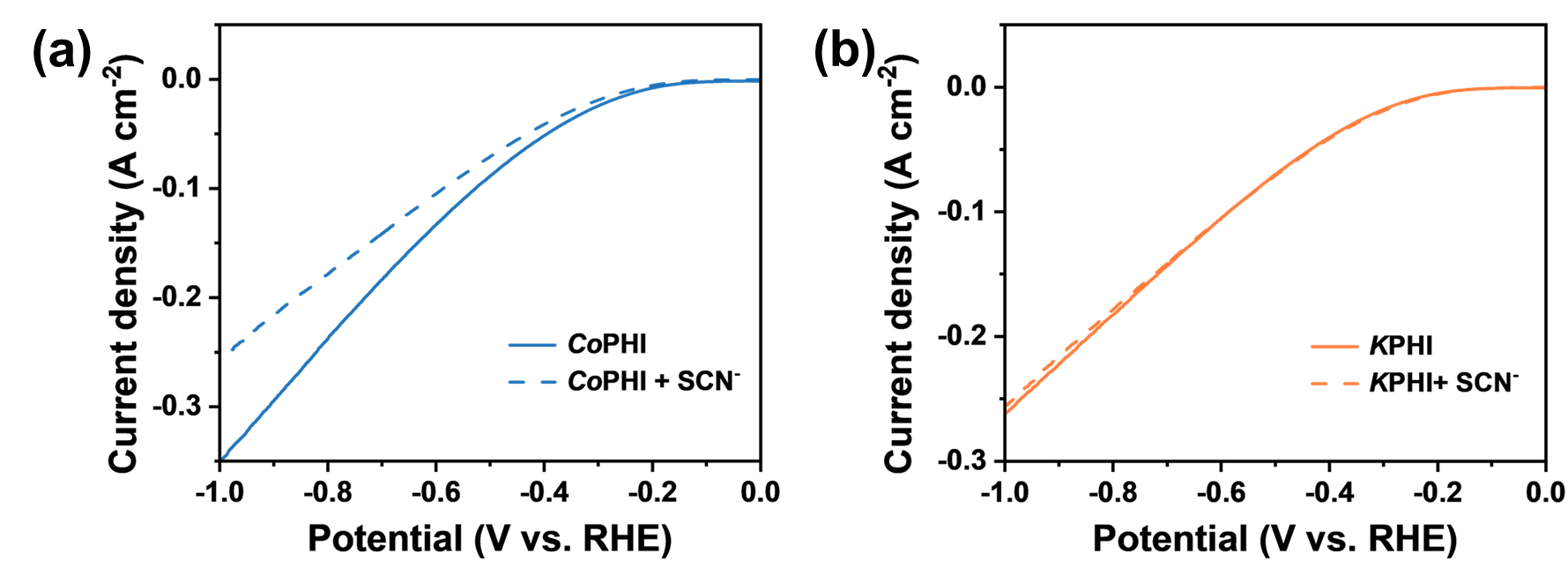


**Figure S19.** LSV curves of (a) *Co*PHI and (b) *K*PHI before and after poisoning with KSCN in 0.1 M KOH with 1.0 M KNO_2_.


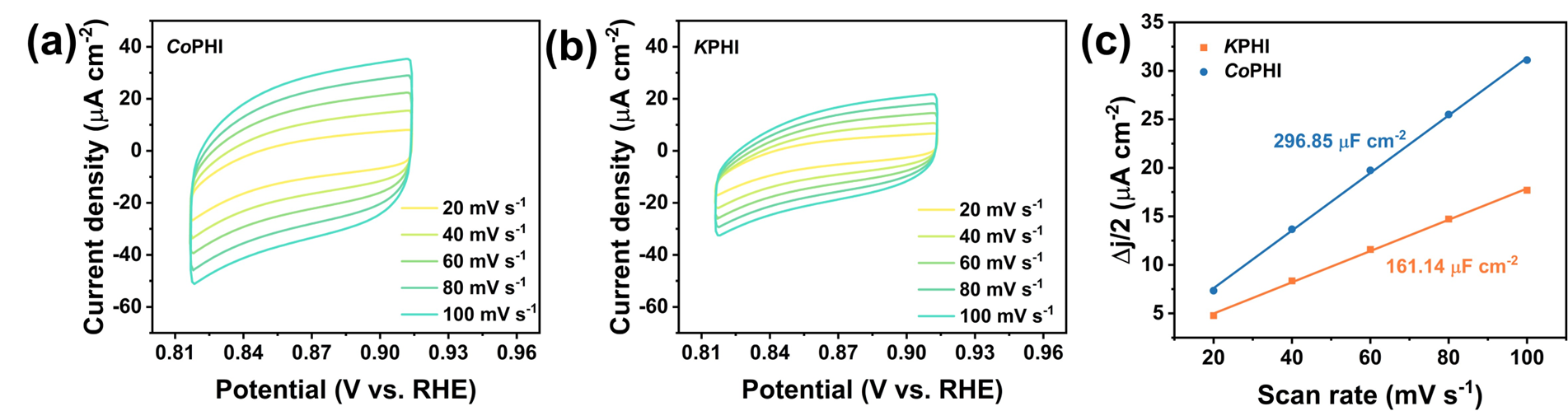


**Figure** **S20.** CV curves with various scan rates at potential window of 0.815−0.915 V vs. RHE of (a) *Co*PHI, (b) *K*PHI. (c) The linear fitting of capacitive currents of *Co*PHI and *K*PHI electrode.


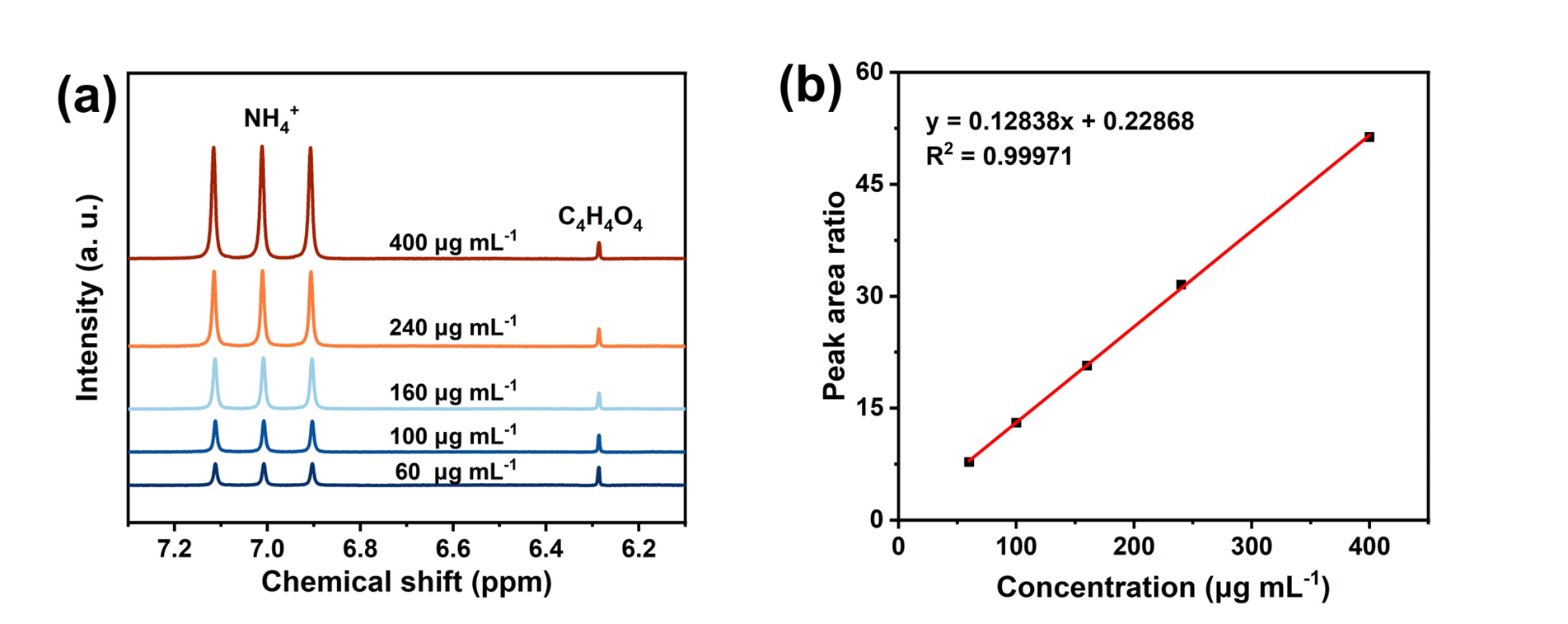


**Figure** **S21.** (a) The ^1^H NMR spectra and (b) standard curves of ^14^NH_4_^+^ in various concentrations with the internal standard of C_4_H_4_O_4_.

**
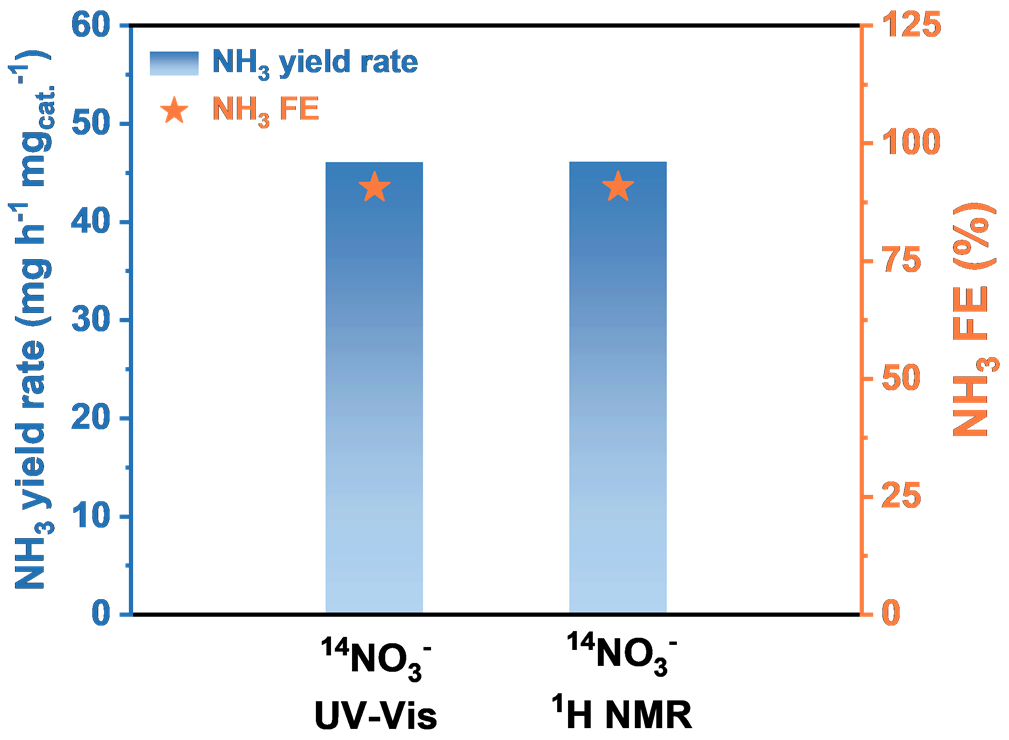
**

**Figure** **S22.** Comparison of the NH_3_ yield rate and FE at -0.8 V vs. RHE in 0.1 M KOH and 1.0 M KNO_3_ calculated by NMR and UV-vis methods.


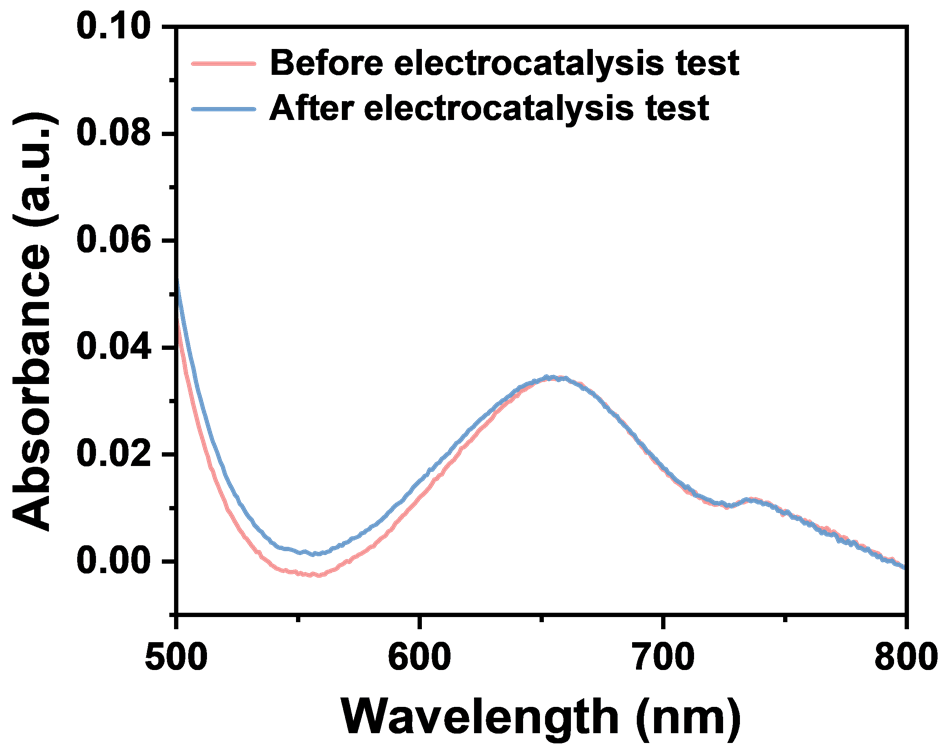


**Figure S23.** UV-vis absorption spectra of *Co*PHI electrolyte before and after electrolysis in 0.1 M KOH solution.


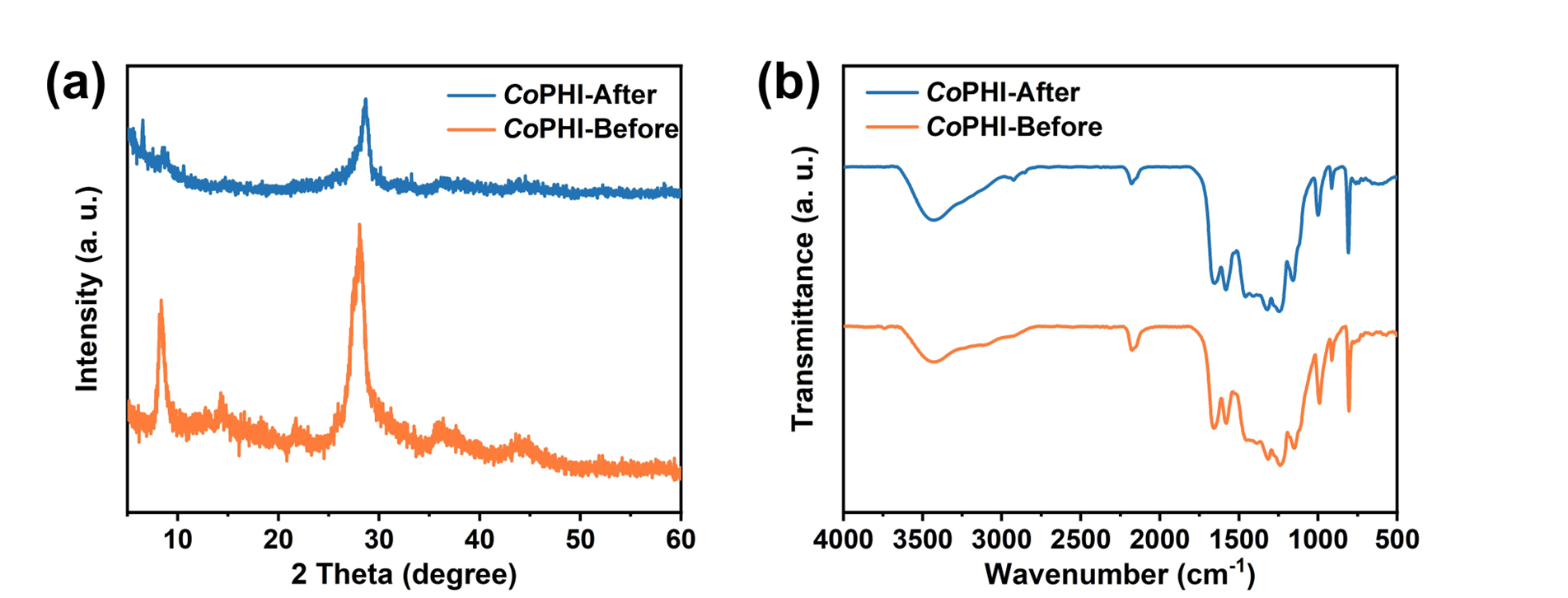


**Figure** **S24.** (a) XRD patterns and (b) FT-IR spectra of *Co*PHI before and after electroreduction.


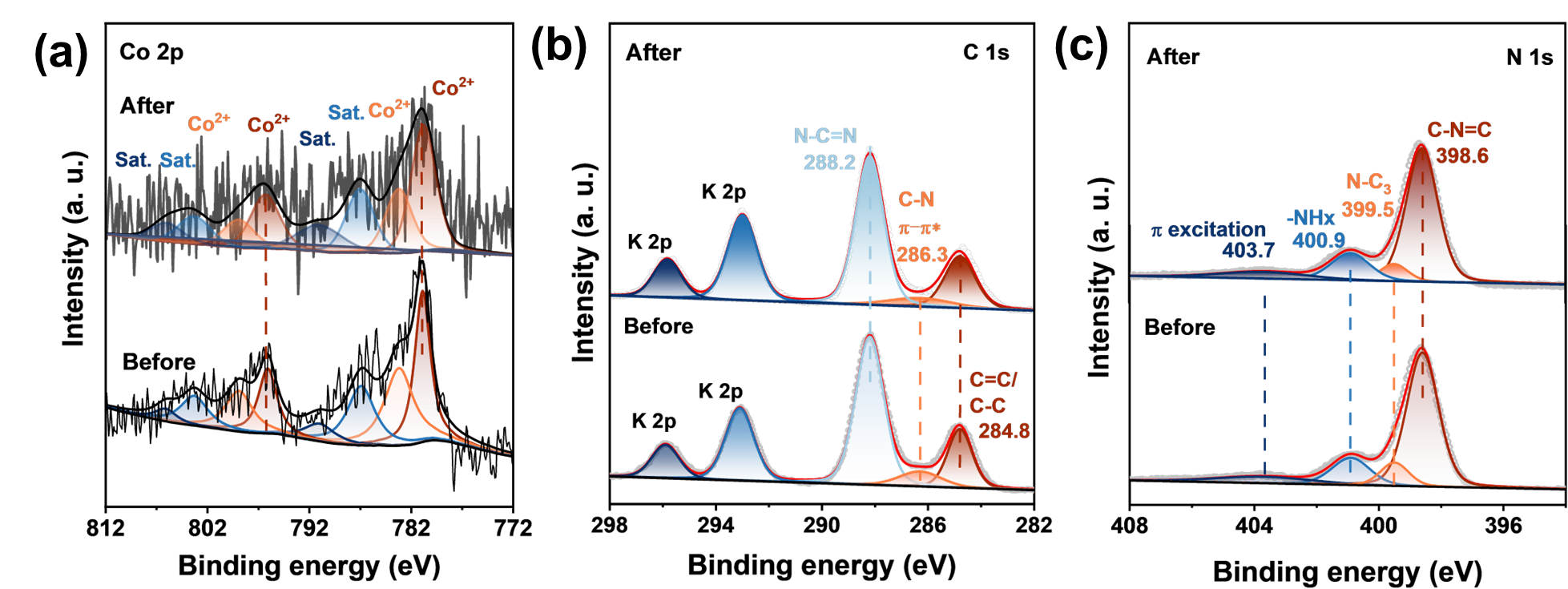


**Figure** **S25.** (a) Co 2p, (b) C 1s, (c) N 1s XPS spectra for *Co*PHI before and after electroreduction.


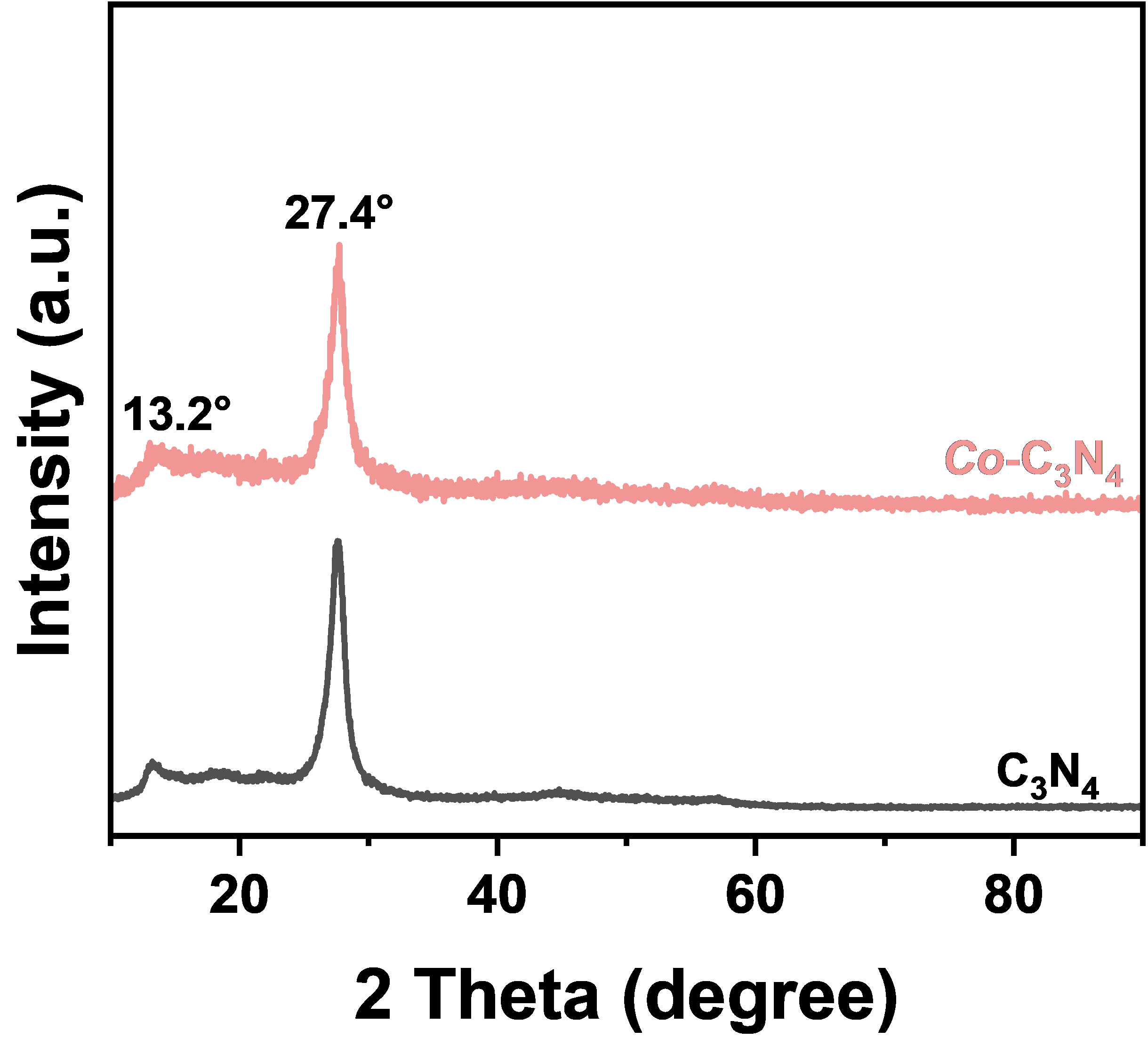


**Figure** **S26**. XRD patterns of C_3_N_4_ and *Co-*C_3_N_4_.


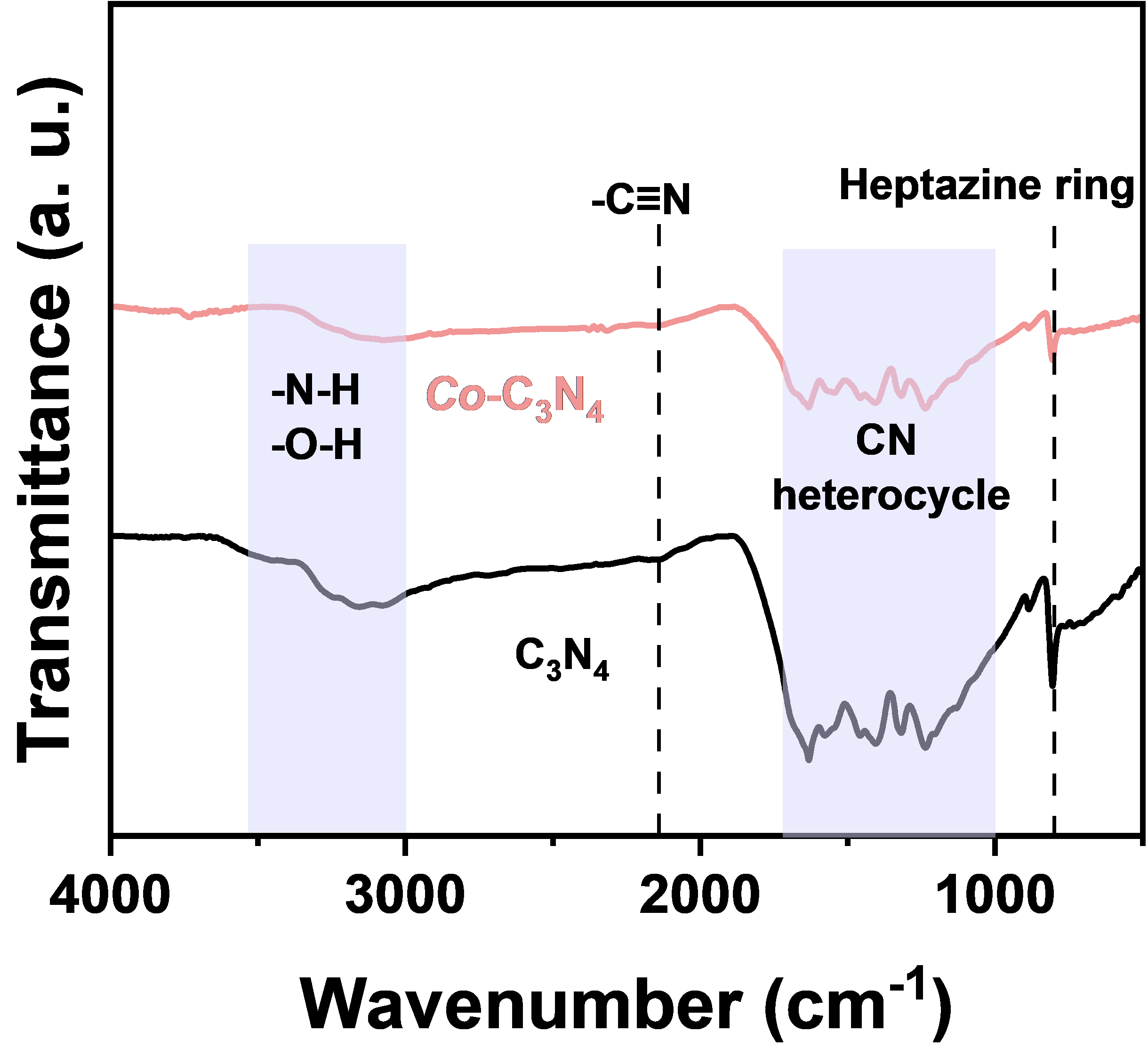


**Figure** **S27**. FT-IR spectra of C_3_N_4_ and *Co*-C_3_N_4_.


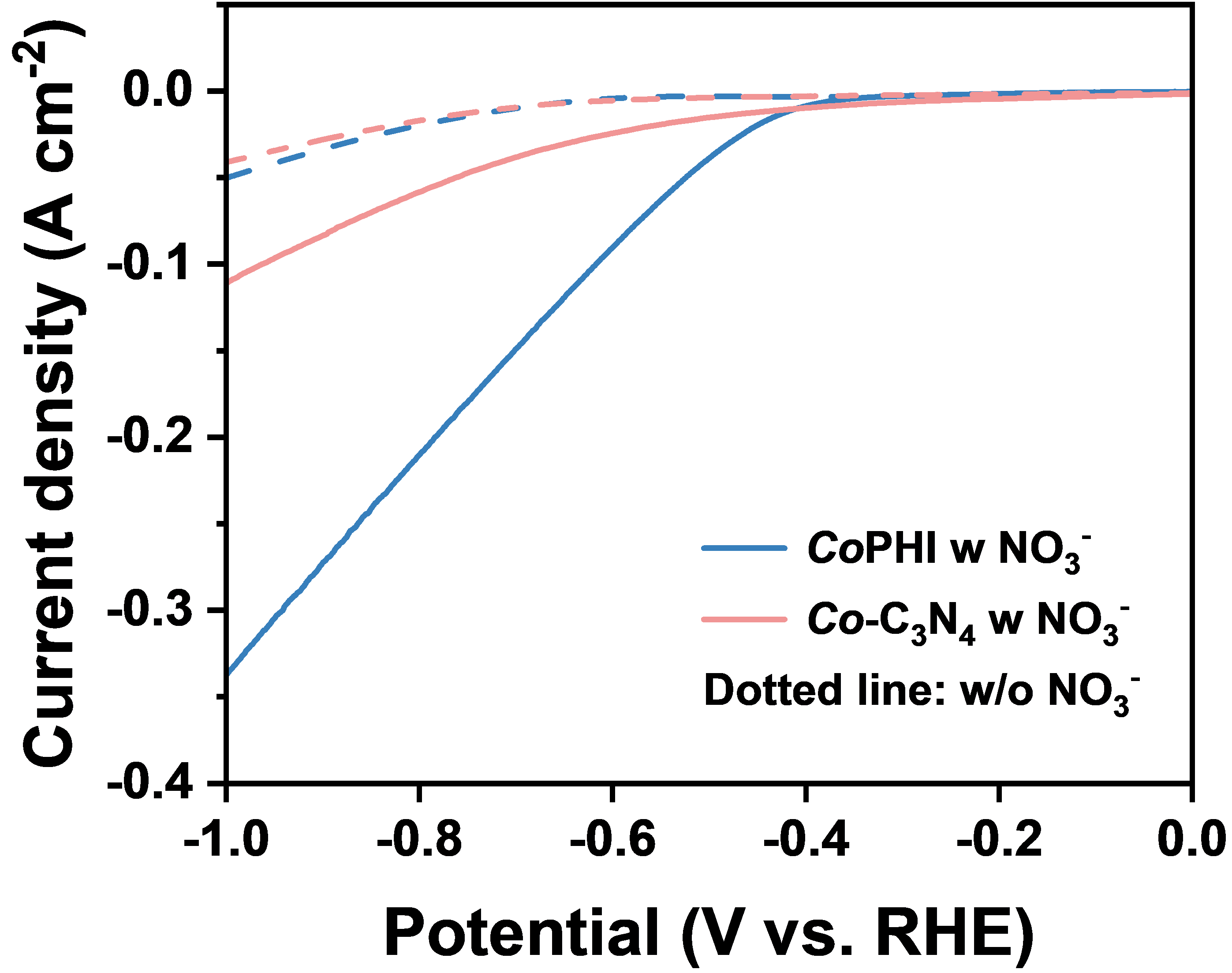


**Figure** **S28**. LSV curves of *Co*PHI and *Co*-C_3_N_4_ in 0.1 M KOH with (solid line) or without (dotted line) 1.0 M KNO_3_.


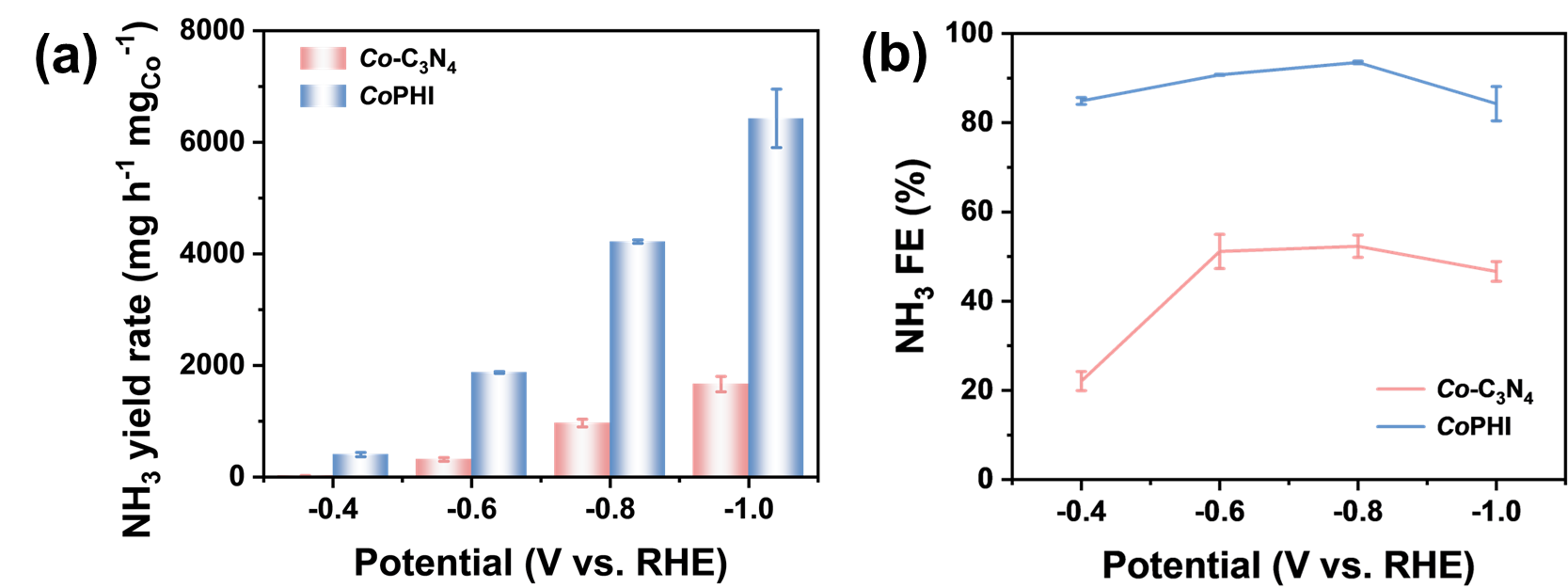


**Figure** **S29**. (a) NH_3_ yield rate and (b) FE of *Co*-C_3_N_4_ and *Co*PHI at different potentials.


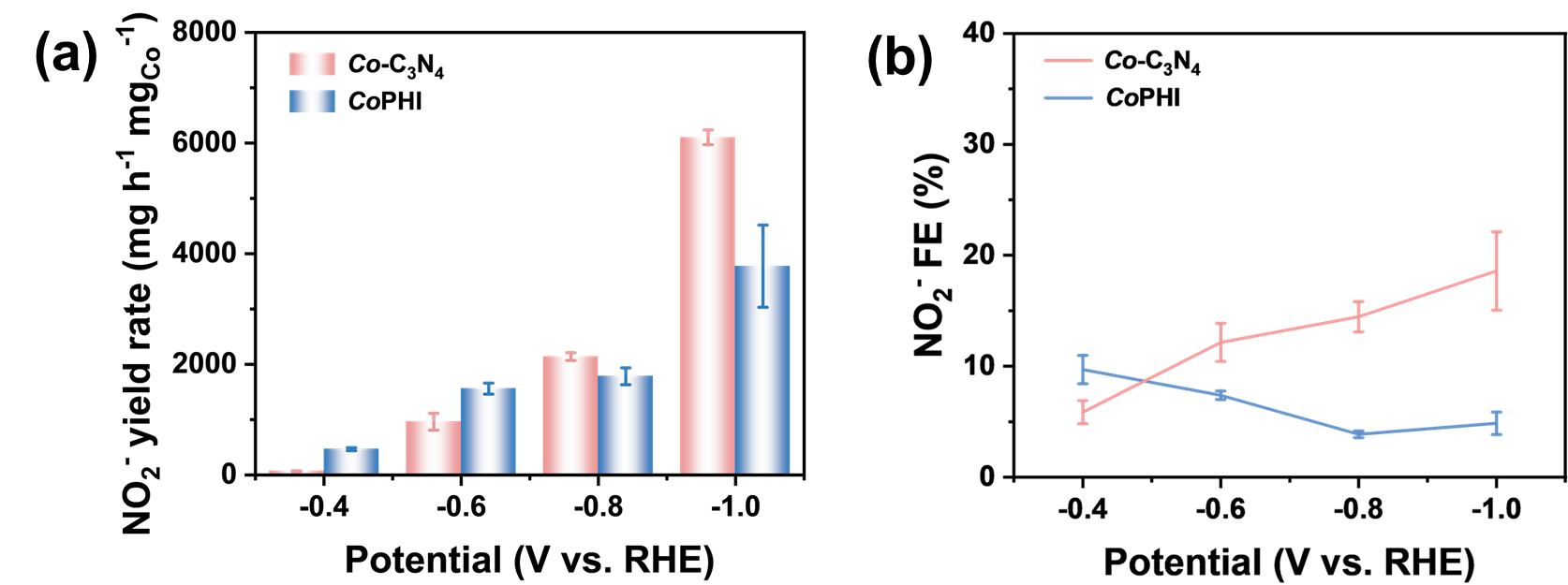


**Figure** **S30**. (a) NO_2_^-^ yield rate and (b) FE of *Co*-C_3_N_4_ and *Co*PHI at different potentials.


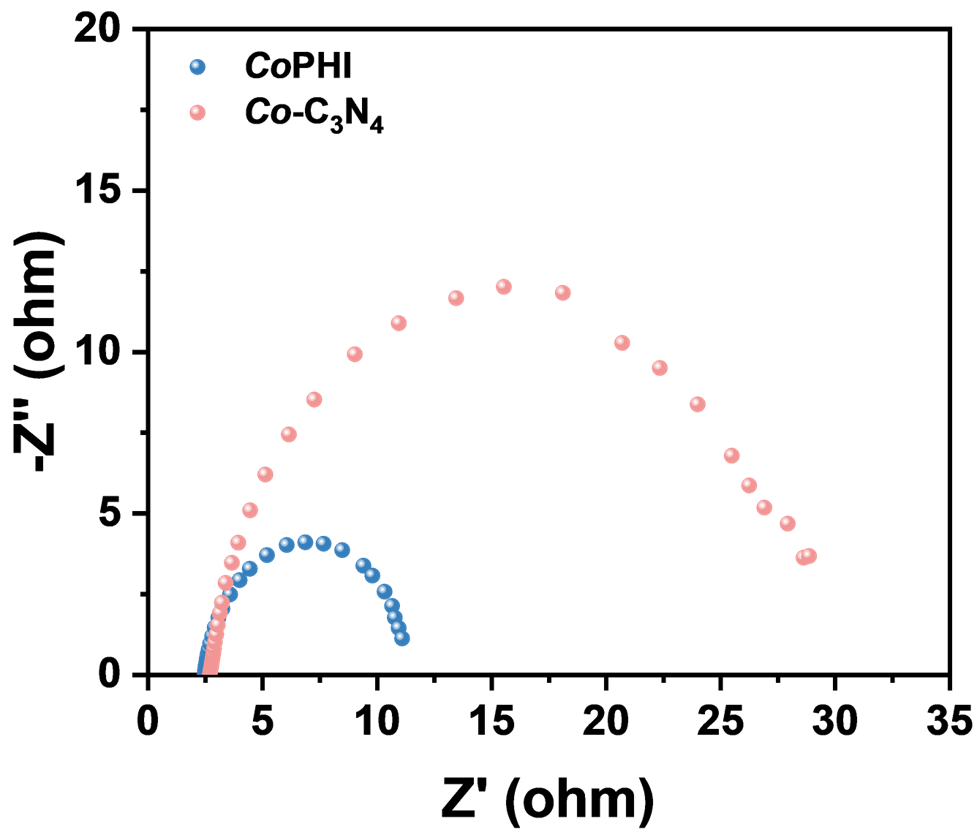


**Figure S31**. EIS curves of *Co*PHI and *Co*-C_3_N_4_ at -0.4 V vs. RHE.


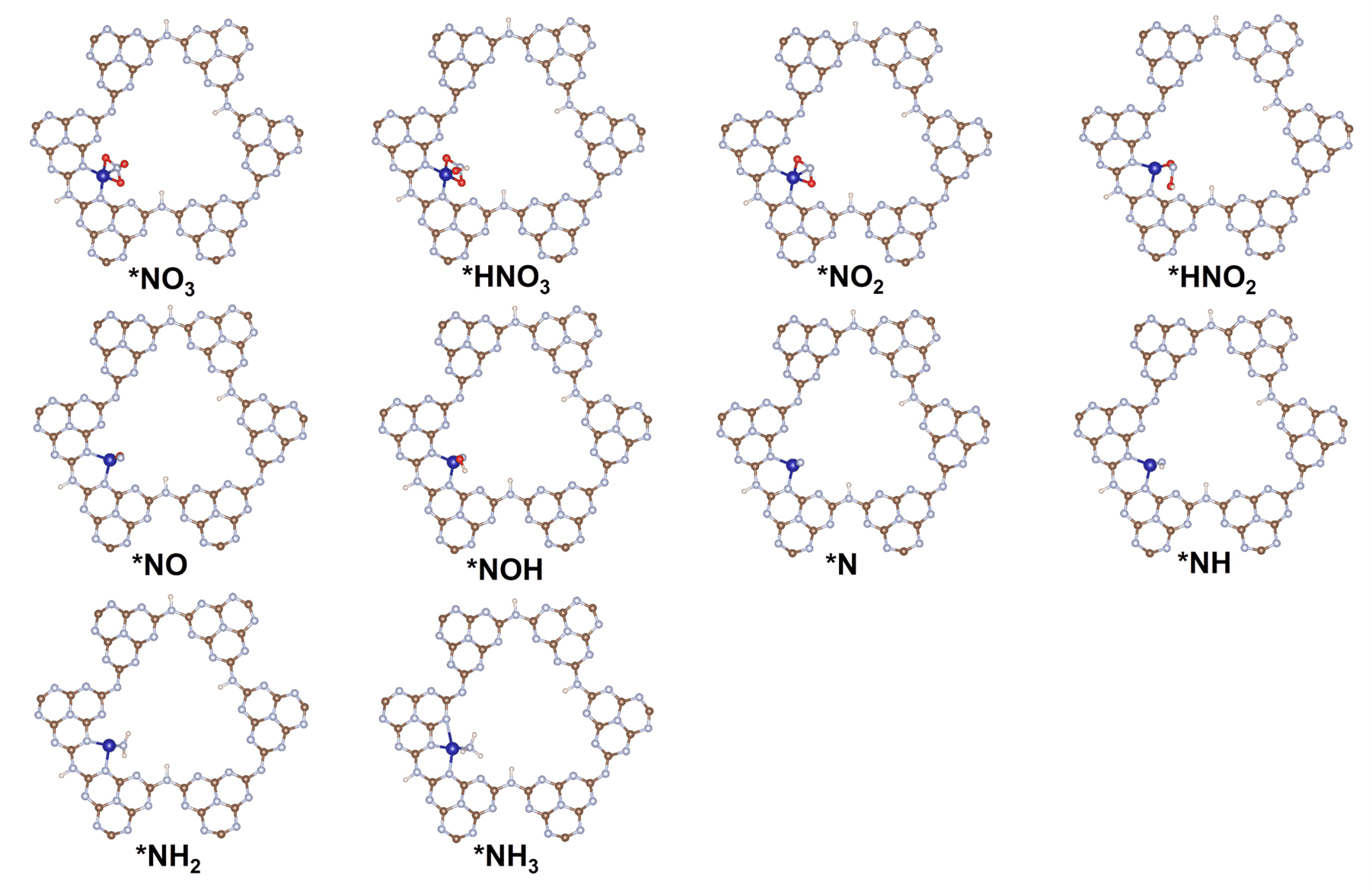


**Figure S32.** Adsorption configurations of different intermediates on *Co*PHI.

**
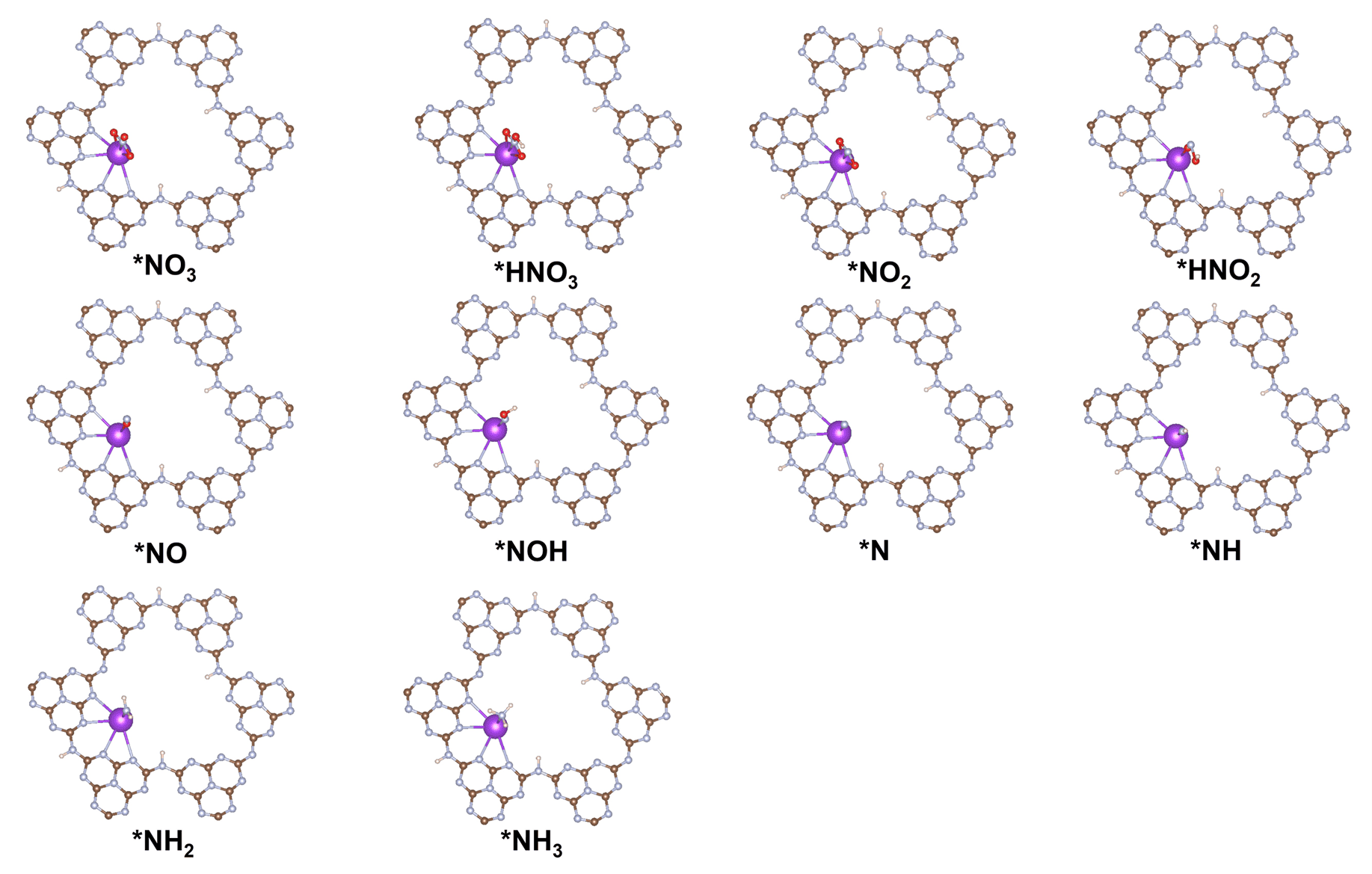
**

**Figure** **S33.** Adsorption configurations of different intermediates on *K*PHI.


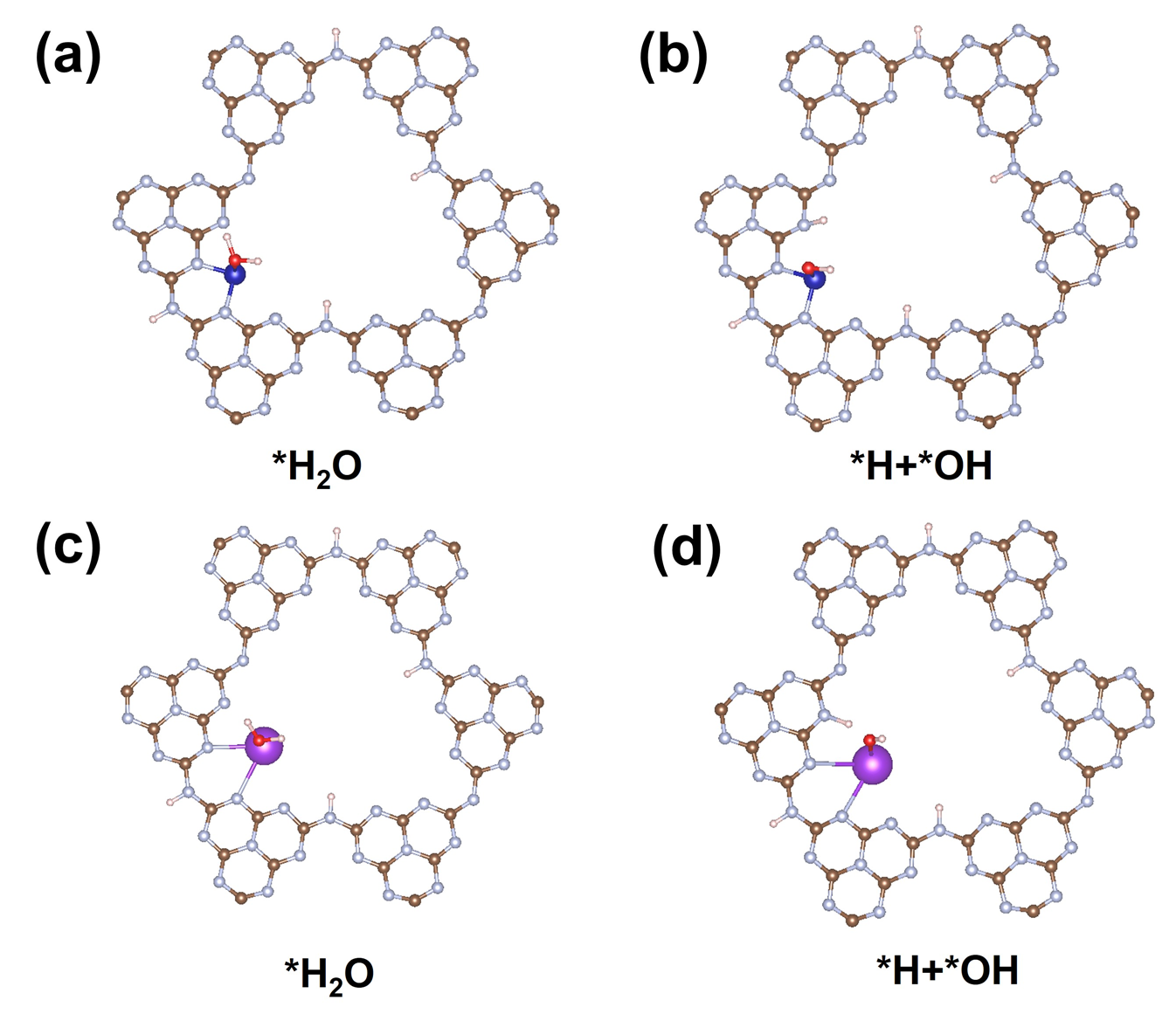


**Figure** **S34.** Optimized Structure diagrams of intermediate adsorption during water splitting on the surfaces of (a-b) *Co*PHI and (c-d) *K*PHI.


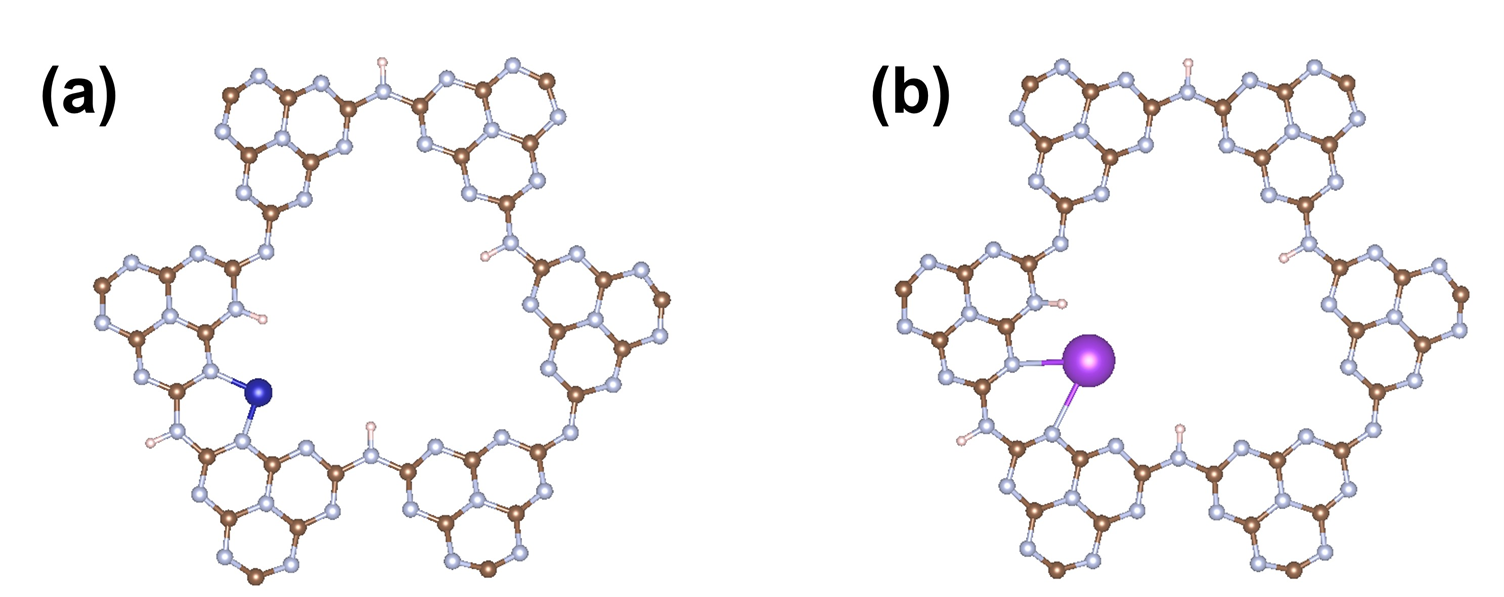


**Figure** **S35.** Adsorption structure diagrams of *H during the HER process on the surfaces of (a) *Co*PHI and (b) *K*PHI.

***References*:**

[1] P. Gao, Z. H. Xue, S. N. Zhang, D. Xu, G. Y. Zhai, Q. Y. Li, J. S. Chen, X. H. Li, *Angew. Chem. Int. Ed.* **2021**, *60*, 20711-20716.

[2] Y. L. Fu, X. T. Yang, Y. Yu, K. Zhou, X. Y. Ye, A. X. Zhang, X. J. Hou, B. B. Chen, F. Q. Fan, Y. H. Li, Y. Fu, *Nano Res.* **2025**, *18*, 94907038.

[3] H. Yin, F. Dong, H. Su, Z. Zhuang, Y. Wang, D. Wang, Y. Peng, J. Li, *ACS Nano* **2023**, *17*, 25614-25624.

[4] Z. Ke, D. He, X. Yan, W. Hu, N. Williams, H. Kang, X. Pan, J. Huang, J. Gu, X. Xiao, *ACS Nano* **2023**, *17*, 3483-3491.

[5] Z. Y. Wu, M. Karamad, X. Yong, Q. Huang, D. A. Cullen, P. Zhu, C. Xia, Q. Xiao, M. Shakouri, F. Y. Chen, J. Y. Kim, Y. Xia, K. Heck, Y. Hu, M. S. Wong, Q. Li, I. Gates, S. Siahrostami, H. Wang, *Nat. Commun.* **2021**, *12*, 2870.

[6] X. Long, F. Huang, T. Zhong, H. Zhao, P. Li, J. Fang, S. Tian, D. Shu, C. He, *Environ. Sci. Technol.* **2025**, *59*, 8555-8567.

[7] L. Liu, T. Xiao, H. Fu, Z. Chen, X. Qu, S. Zheng, *Appl. Catal. B: Environ.* **2023**, *323*, 122181.

[8] J. Guan, L. Cai, W. Li, H. Zhou, Y. Huang, *Appl. Catal. B: Environ.* **2024**, *358*, 124387.

[9] X. F. Cheng, J. H. He, H. Q. Ji, H. Y. Zhang, Q. Cao, W. J. Sun, C.-L. Yan, J. M. Lu, *Adv. Mater.* **2022**, *34*, 2205767.

[10] S. Lu, G. Lin, H. Yan, Y. Li, T. Qi, Y. Li, S. Liang, L. Jiang, *ACS Catal.* **2024**, *14*, 14887-14894.

[11] L. Zhong, Q. Chen, H. Yin, J. S. Chen, K. Dong, S. Sun, J. Liu, H. Xian, T. Li, *Chem. Commun.* **2023**, *59*, 8973-8976.

[12] Y. Lv, S. W. Ke, Y. Gu, B. Tian, L. Tang, P. Ran, Y. Zhao, J. Ma, J.-L. Zuo, M. Ding, *Angew. Chem. Int. Ed.* **2023**, *62*, e202305246.

[13] X. Fan, J. Liang, L. Zhang, D. Zhao, L. Yue, Y. Luo, Q. Liu, L. Xie, N. Li, B. Tang, Q. Kong, X. Sun, *Carbon Neutral.* **2022**, *1*, 6-13.

[14] J. Q. Ni, J. Yan, F. H. Li, H. F. Qi, Q. Z. Xu, C. L. Su, L. K. Sun, H. L. Sun, J. Ding, B. Liu, *Adv. Energy Mater.* **2024**, *14*, 2400065.

[15] H. Fu, Y. Chen, H. Huang, C. Chen, T. Zhu, F. Lai, N. Zhang, T. Liu, *ACS Sustain. Chem. Eng.* **2024**, *12*, 346-354.

[16] Y. Yu, C. Wang, Y. Yu, Y. Wang, B. Zhang, *Sci. China Chem.* **2020**, *63*, 1469-1476.

[17] S. Sun, C. Dai, P. Zhao, S. Xi, Y. Ren, H. R. Tan, P. C. Lim, M. Lin, C. Diao, D. Zhang, C. Wu, A. Yu, J. C. J. Koh, W. Y. Lieu, D. H. L. Seng, L. Sun, Y. Li, T. L. Tan, J. Zhang, Z. J. Xu, Z. W. Seh, *Nat. Commun.* **2024**, *15*, 260.

[18] M. Liu, J. Qi, G. Luo, Y. Cui, W. Wei, W. Chen, Y. Zhang, Y. Wen, X. Wang, R. Wang, *Adv. Funct. Mater.* **2025**, e23666.

[19] Y. Ren, J. Wang, L. Yang, Q. Gao, P. Li, Y. Zhang, Y. Xiong, Z. Fan, X. Quan, Y. Liu, *Environ. Sci. Technol.* **2025**, *59*, 11414-11425.

[20] J. Yang, H. Qi, A. Li, X. Liu, X. Yang, S. Zhang, Q. Zhao, Q. Jiang, Y. Su, L. Zhang, J. F. Li, Z. Q. Tian, W. Liu, A. Wang, T. Zhang, *J. Am. Chem. Soc.* **2022**, *144*, 12062-12071.

[21] J. Guo, L. H. Zhang, Y. Guo, C. Shi, A. Han, D. Wang, F. Yu, *Angew. Chem. Int. Ed.* **2026**, *65*, e16401.

[22] W. Zhang, Y. Zhou, Y. Zhu, Y. Guo, B. Zhang, L.-H. Zhang, F. Li, F. Yu, *Small* **2024**, *20*, 2404792.

[23] Y. Wang, W. Zhang, W. Wen, X. Yu, Y. Du, K. Ni, Y. Zhu, M. Zhu, *Adv. Funct. Mater.* **2023**, *33*, 2302651.

[24] Y. T. Xu, M. Y. Xie, H. Zhong, Y. Cao, *ACS Catal.* **2022**, *12*, 8698-8706.

[25] Y. Xue, Q. Yu, Q. Ma, Y. Chen, C. Zhang, W. Teng, J. Fan, W. x. Zhang, *Environ. Sci. Technol.* **2022**, *56*, 14797-14807.

[26] Z. Cui, H. Wang, C. Li, W. Peng, J. Liu, *ACS Nano* **2025**, *19*, 31050-31064.
